# Endothelial-related gene expression profile of muscle adaptation to high-intensity interval training in arterial hypertension

**DOI:** 10.1371/journal.pone.0358696

**Published:** 2026-10-14

**Authors:** Helen Louisi Camilo, Natália Zamberlan Ferreira, Luciana Sanae Ota, Antônio Cláudio Bongiovani, Ines Cristina Giometti, Aline de Oliveira Santos, Paula Paccielli Freire, Katashi Okoshi, Rafael S. Floriano, Ivan J. Vechetti, Dijon Henrique Salomé de Campos, Marina Politi Okoshi, Francis Lopes Pacagnelli

**Affiliations:** 1 Department of Medicine, University of Western São Paulo, UNOESTE, Presidente Prudente, São Paulo, Brazil; 2 Health Science Program, University of Western São Paulo, UNOESTE, Presidente Prudente, São Paulo, Brazil; 3 Animal Science Program, University of Western São Paulo, UNOESTE, Presidente Prudente, São Paulo, Brazil; 4 Faculties of Dracena, UNIFADRA, Dracena, São Paulo, Brazil; 5 Department of Immunology, Institute of Biomedical Sciences, University of São Paulo, USP, São Paulo, Brazil; 6 Experimental Research Unity, Faculty of Medicine, São Paulo State University, UNESP, Botucatu, São Paulo, Brazil; 7 Department of Nutrition and Health Sciences, University of Nebraska-Lincoln, Lincoln, Nebraska, United States of America; Hamad Bin Khalifa University, QATAR

## Abstract

Hypertension and aging are associated with endothelial dysfunction in skeletal muscle, potentially compromising exercise capacity. Although high-intensity interval training (HIIT) has been proposed as an effective non-pharmacological strategy for individuals with hypertension, its effects on endothelial-related markers in aging skeletal muscle remain unclear This study investigated the impact of HIIT on endothelial-related gene expression in hypertensive aging rats. Twenty male 12-month-old spontaneously hypertensive rats were assigned to sedentary (n = 10) or trained (n = 10) groups. The training protocol (50 min/day, 5 times/week) was performed for 8 weeks, on a treadmill adapted for rodents. Gene expression of nitric oxide synthase 3 (*NOS3*), vascular endothelial growth factor (*VEGF*), endothelin-1 (*EDN1*), and phosphoinositide-3-kinase regulatory subunit 2 (*PIK3R2*) was assessed by reverse transcription quantitative polymerase chain reaction and by reanalysis of transcriptomic datasets available in the Gene Expression Omnibus (GSE109657 and GSE163356). Eight weeks of HIIT reduced blood pressure and improved physical performance, without altering the expression of *NOS3, VEGF, EDN1*, and *PIK3R2* in the soleus and diaphragm muscles. These findings are consistent with an independent transcriptomic dataset from vastus lateralis biopsies of healthy subjects performing HIIT protocols (4 times/week for 6 weeks); however, changes were identified in *NOS3*, *VEGF,* and *PIK3R2* genes in the vastus lateralis muscle after an HIIT protocol performed 3 times/week for 3 weeks. Collectively, the results indicate that HIIT improves functional capacity and blood pressure regulation in hypertensive aging rats, without concomitant changes in endothelial-related gene expression. These results are comparable with those found in healthy individuals undergoing a similar period of HIIT.

## 1. Introduction

Hypertension is a chronic disease and its pathophysiology involves complex interactions between environmental and behavioral factors, genes, hormonal networks, and multiple organ systems [1]. An estimated 1.4 billion adults aged 30–79 years worldwide had hypertension in 2024; representing 33% of the population in this age range [2]. With aging, systolic blood pressure levels tend to rise steadily to the end of life [3]. Therefore, the incidence of hypertension increases with age, and it is estimated that an individual over the age of 50 with normal arterial pressure has a high likelihood of becoming a hypertensive patient in the next two decades, due to the increase in arterial stiffness related to the deterioration of elastic elements [4]. The increase in peripheral vascular resistance in hypertension alters the properties of muscular vascular structures, damaging the transport and permeability of the nutritional and energetic contribution of muscles, leading to impaired contractile function and loss of physical activity capacity [5–8]. Thus, vascular remodeling is associated with aging and its effects are reinforced by hypertension [7,9].

Hypertension is characterized by deregulation of multiple biological pathways, including those related to inflammation, oxidative stress, renal function, the sympathetic nervous system, and vascular tone. Endothelial dysfunction and arterial remodeling are hallmarks of hypertension [10]. Modifications in the gene expression of factors derived from the endothelium and vascular smooth muscle can profoundly alter blood pressure control, as these genes regulate proteins that directly affect vascular tone, peripheral resistance, and vessel structure. The relationship between arterial hypertension and endothelial dysfunction is bidirectional, with each condition reinforcing the severity of the other. Endothelial cells play a central role in the pathogenesis of hypertension. Similarly, increased blood pressure promotes endothelial dysfunction and contributes to a prothrombotic and proinflammatory status, thereby increasing cardiovascular risk [11]. Endothelial dysfunction and reduced nitric oxide (NO) production by endothelial nitric oxide synthase have also been implicated in the pathogenesis of hypertension [12]. *NOS3* encodes the enzyme nitric oxide synthase in the endothelium, which is responsible for the production of NO, through an oxygen-dependent reaction. NO modulates vascular endothelial growth factor (VEGF) activity by binding to its receptors and initiating signaling cascades that lead to the production of NO and angiogenic activation of endothelial cells [13]. Hypertensive individuals presented 36% lower levels of VEGF protein and 22% lower capillary density in skeletal muscle [14]. In addition, phosphoinositide-3-kinase regulatory subunit 2 (*PIK3R2)* may contribute to hypertension through impaired PI3K/Akt signaling, causing endothelial dysfunction and altered angiogenesis. The effects of hypertension and aging are exacerbated when considered together, and result in a reduction in the Akt signaling pathway [9].

Other mechanisms linked to endothelial dysfunction include the increased expression of Endothelin-1 (*EDN-1*), that activates specific areas in the central nervous system, resulting in an increase in sympathetic tone and/or a greater release of vasoconstrictor hormones, as well as potentiating the action of other vasoconstrictor substances [13]. In addition to these central effects, increased expression of the *EDN1* gene disrupts the balance between vasodilatory and vasoconstrictor factors, promotes vascular inflammation, increases oxidative stress, and contributes to vascular remodeling. These effects occur because the peptide endothelin-1 (ET-1) is directly involved in the regulation of vascular tone and peripheral vascular resistance, as it can decrease NO production, increase NO degradation, and induce a pro-inflammatory phenotype in endothelial cells [15]. Elevated ET-1 levels have also been reported in skeletal muscle and plasma in aging and hypertensive individuals, and physical activity may counteract these negative markers [16]. The aforementioned mechanisms contribute to increased peripheral vascular resistance and directly affect blood pressure levels. In addition, the interaction between hypertension and aging could be responsible for the acceleration of vascular remodeling [7,9].

One of the proposed treatments for hypertension which has been gaining interest in the scientific community is High-Intensity Interval Training (HIIT). HIIT has been demonstrated to be effective in reducing fat mass, improving exercise tolerance and reducing blood pressure in older treated hypertensive individuals. This training method alternates periods of high-intensity aerobic exercise with moderate-low intensity passive or active recovery [17–20]. Moreover, HIIT can be beneficial from the perspective of public health, as “lack of time” is a notable barrier for adhering to a physical training program in a great part of the population [21,22]. Physical exercise can improve endothelial function and modulate angiogenic biomarkers in hypertensive patients. Evidence from a recent systematic review and meta-analysis demonstrated that high-intensity aerobic exercise had the most notable intervention effect on nitric oxide levels, while moderate intensity resistance exercise had the strongest effect on plasma endothelin-1 concentrations [23]. However, to date, these effects have been predominantly described based on assessments in plasma, systemic compartments, or vascular tissues [8,23,24]. In contrast, the local effects of HIIT on gene expression of skeletal muscle markers is relevant given the central role of skeletal muscle in the regulation of peripheral vascular resistance [14]. Considering that endothelial dysfunction and arterial remodeling are essential characteristics of hypertension, and that endothelium-derived bioactive substances contribute to the maintenance of vascular tension, endothelial integrity, and blood flow stability, it is important to understand these biomarkers in a more cost-effective exercise modality, such as HIIT. Better understanding of these effects may support the prescription of HIIT for the prevention and treatment of hypertension in older adults.

Based on these considerations, we hypothesized that HIIT enhances pro-angiogenic and vasodilatory signaling, thereby improving peripheral microcirculation in aging hypertension. Accordingly, the aim of the current study was to evaluate the effects of HIIT on molecular markers of skeletal muscle microcirculation in spontaneously hypertensive aged rats. Specifically, we assessed the expression of genes encoding proteins involved in vasomotor regulation (*NOS3* and *EDN1*) and angiogenesis (*VEGF* and *PIK3R2*) in the soleus and diaphragm muscles. To strengthen the translational relevance of our findings, we further analyzed the expression of these genes in publicly available datasets from individuals who underwent HIIT (GSE109657 and GSE163356).

## 2. Materials and methods

### 2.1. Animals

All the experimental procedures involved in this study were approved by the Ethics Committee for Animal Use of the University of Western São Paulo under protocols n. 3971 and 3975, comply with the principles of animal care in laboratories formulated by the Brazilian Society of Science in Laboratory Animals (SBCAL), and are in accordance with the Guide for Care and Use of Laboratory Animals, published by the National Research Council (NIH, revised in 2011) [25].

We used 20 male Spontaneously Hypertensive Rats (SHR, twelve-months of age, obtained from the Central Animal Hospital of the São Paulo Federal University (Unifesp), São Paulo, SP, Brazil. The rats were randomly divided through an Excel-generated sequence into two groups: sedentary control spontaneously hypertensive rats (SHR-SED, n = 10) and trained spontaneously hypertensive rats (SHR-HIIT, n = 10). Throughout the experimental period, the rats were housed in plastic boxes, divided into four rats in each box, with a temperature range from 21–23°C and relative air humidity from 50–60%. The animals were taken out of the boxes for the training and returned after. The rats received food (Supralab, Alisul R, Brazil) and water *ad libitum.* The light-dark cycle (inverted 12-hour cycle- light from 7 pm to 7 am) was controlled.

### 2.2. Experimental design

The rats were subjected to HIIT for 8 weeks. Both before starting and after completing the physical training protocol, systolic blood pressure and maximum exercise capacity were evaluated. An additional test of maximum exercise capacity was performed in order to adjust the load at the end of the fourth week in the animals subjected to HIIT. At the end of 8 weeks of HIIT, animals were euthanized, soleus and diaphragm muscles were snap-frozen in liquid N_2_ and stored at –80°C for gene expression analysis (Fig 1).

**Fig 1:**
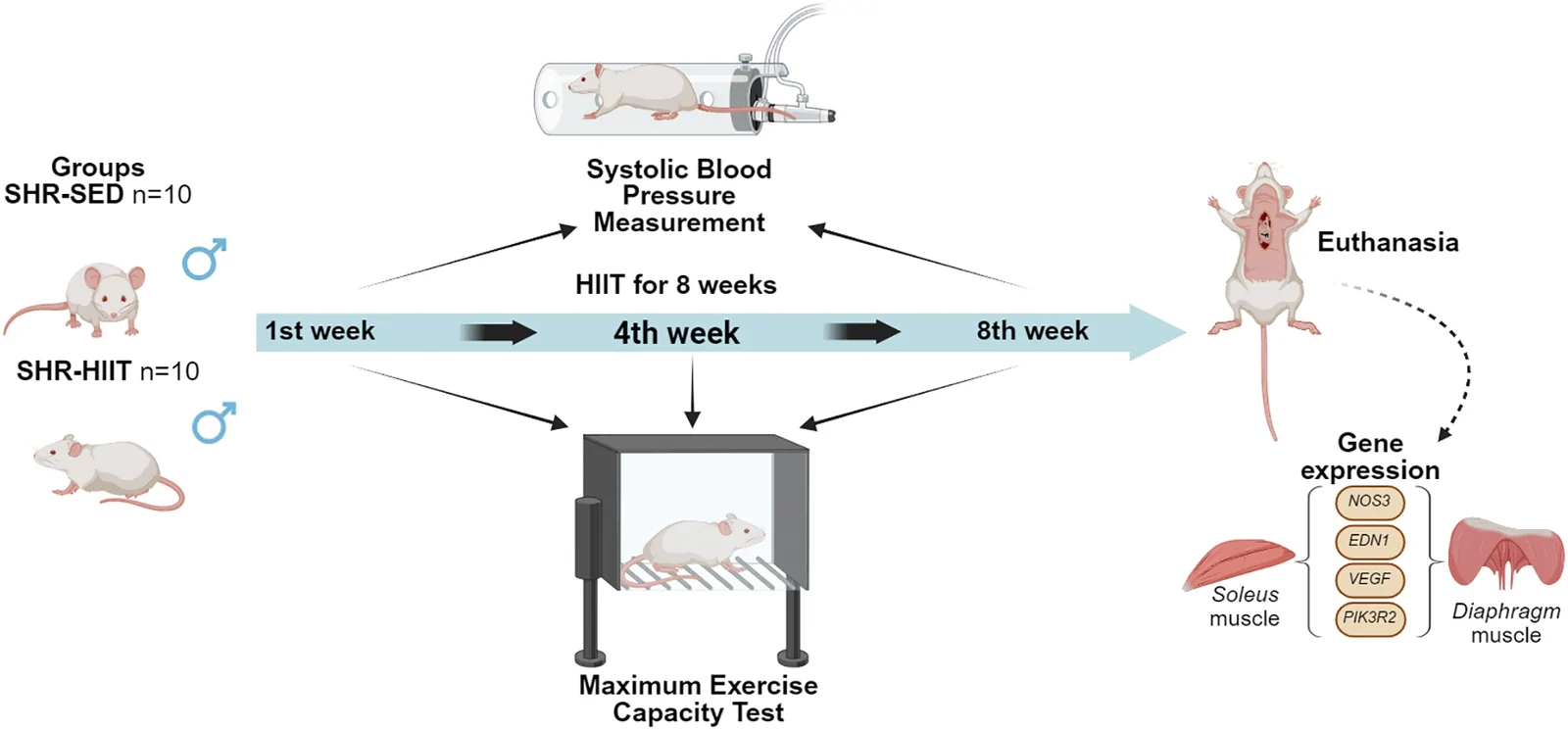
Experimental design. SHR-SED: Spontaneously Hypertensive Sedentary Rats; SHR-HIIT: Spontaneously Hypertensive Rats subjected to HIIT; HIIT: High-Intensity Interval Training; *NOS3:* nitric oxide synthase 3; *VEGF:* vascular endothelial growth factor; *EDN1:* endothelin-1; *PIK3R2:* phosphoinositide-3-kinase regulatory subunit 2.

### 2.3. Systolic blood pressure (SBP)

To assess blood pressure control, systolic blood pressure was measured 48 hours after the maximum exercise capacity test and 72 hours after the final HIIT protocol session, at the same time of day, during the dark phase of the inverted light cycle (8–9 a.m.). Plethysmography was employed, using the tail-cuff method (Narco Bio-System^®^, model 709−0610, International Biomedical, Inc., USA). Before the measurements, the animals were maintained at 37°C for 15 minutes to promote dilation of the caudal artery, and the average of two SBP readings was recorded [26–29].

### 2.4. Maximum exercise capacity

Before assessment of maximum exercise capacity, the rats were familiarized with the treadmills (model TK 1 – Inbramed, São Paulo/SP, Brazil) at a velocity of 6 meters/minute, with 0% inclination, for 10 minutes per day over one week. The exercise capacity test began at 6 meters/minute, with the velocity increased by 3 meters/minute every 3 minutes until exhaustion [28,30]. Exhaustion was defined as the inability to continue running despite manual stimulation or loss of coordinated movement. Functional capacity was quantified as total distance, calculated from treadmill velocity and total test duration. This protocol was conducted at baseline, at week 4 for adjustment of training intensity, and at week 8 (24 hours after the final HIIT session) [28].

### 2.5. High-intensity interval training

Training were performed five days per week, over an 8-week period, with a total duration of approximately 50 min per session. The protocol consisted of a high-intensity interval training (HIIT) program adapted and inverted from previously described models [31,32]. Each session included repeated bouts of high-intensity exercise interspersed with periods of active recovery, preceded by a warm-up and followed by a recovery period. The training was performed between 2:00 and 2:50 p.m.

The HIIT phase began at 95% of the maximal velocity achieved during the exhaustion test for 4 minutes, alternated with 65% of maximal velocity for 3 minutes. This cycle was repeated five times during weeks 1 and 2. During weeks 3 and 4, the same intensities were maintained, but the number of repetitions was increased to six or seven consecutive bouts.

Before the start of week 5, a second maximal exercise test was performed to reassess maximal running velocity, and training intensity was adjusted accordingly. During weeks 5 and 6, HIIT was performed at 95% of the maximal velocity obtained in the second exhaustion test, repeated seven times and alternated with 65% of maximal velocity for 3 minutes. Training intensity was further increased by 15% in week 7 and by 18% in week 8, while maintaining the same active recovery intensity (65% of maximal speed for 3 minutes) and seven repetitions. The overall experimental design is summarized in Fig 2.

**Fig 2:**
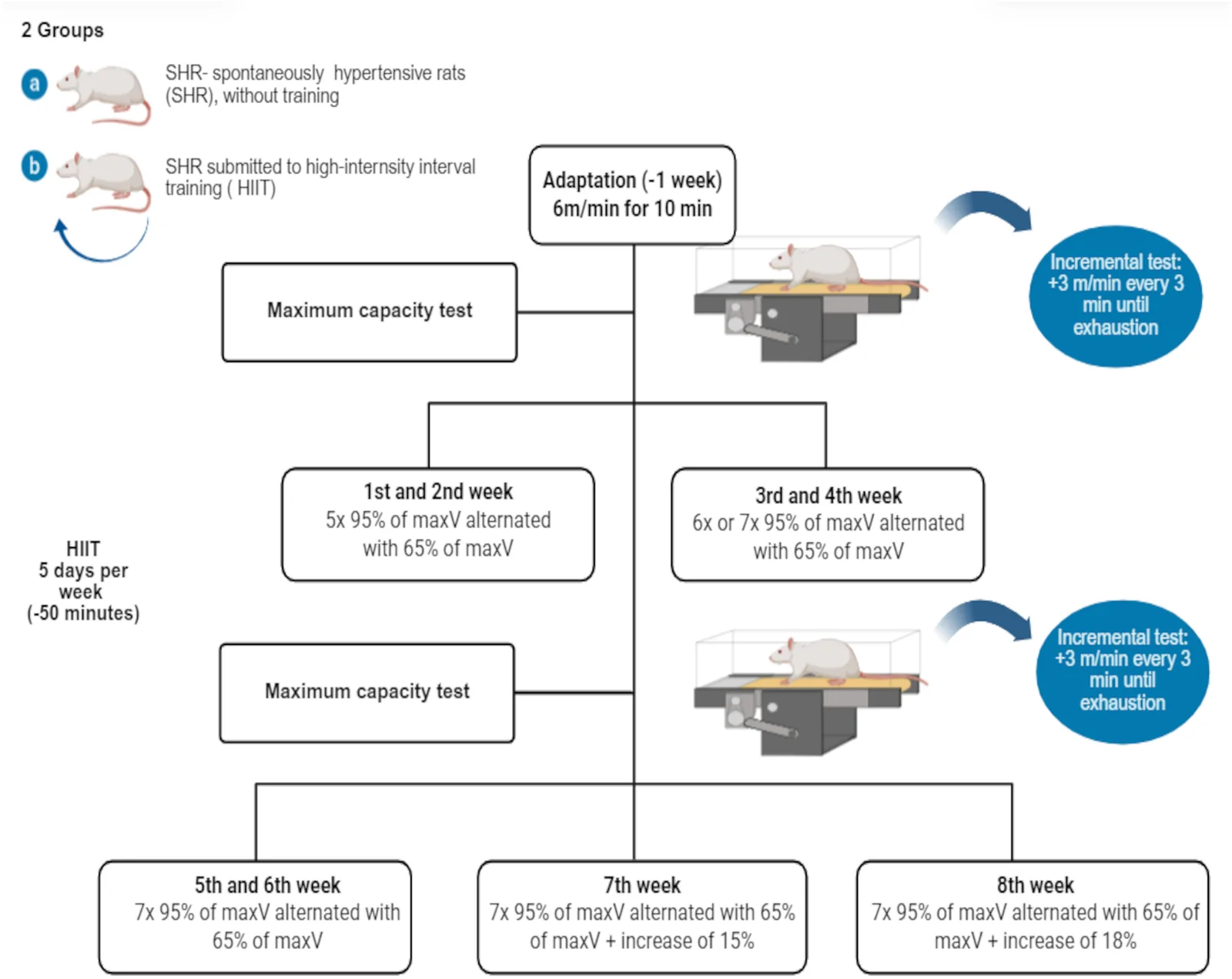
Experimental design and HIIT protocol. Spontaneously hypertensive rats (SHR) were divided into sedentary (SHR-SED) or trained (SHR-HIIT) groups. The high-intensity interval training (HIIT) protocol was performed 5 days/week for 8 weeks, with progressive increases in maximal velocity (maxV) and two maximum capacity tests for load adjustment.

### 2.6. Euthanasia

The rats were subjected to euthanasia using approved methods for their specific species, development stage, and size. Two days (48 hours) after the final HIIT session, the rats were anesthetized with an intramuscular injection of a mix of Ketamine (50 mg/kg ^– 1^; Dopalen^®^) and Xilazine (0.5 mg/kg^–1^; Anasedan^®^) administered by Dr Diego Generoso, veterinarian (Botucatu/ SP, Brazil). The level of anesthesia was assessed by means of relaxation of muscular tonus, relaxation of mandibular tonus, and observance of the foot withdrawal reflex [33]. The rats were then subjected to euthanasia by decapitation, which was performed by an experienced individual (one of the authors of the present study). Tissue samples from skeletal soleus and diaphragm muscles were collected, frozen in liquid nitrogen, and kept at a temperature of −80 °C.

### 2.7. *NOS3*, *EDN1*, *VEGF,* and *PIK3R2* gene expression

Total RNA was isolated from the soleus and diaphragm muscles using Trizol (Invitrogen), according to the manufacturer’s instructions. RNA quantification was performed using a spectrophotometer (Nanodrop, Trinean). The RNA purity was considered satisfactory when the ratio between optical densities of 260 and 280 nm was approximately 2.0. The cDNA reserve transcription kit and RT-PCR (Invitrogen Life Technologies, Carlsbad, United States) were used for the synthesis of 20 μL of complementary DNA (cDNA) from 1000 ng of the total RNA. Next, aliquots of 2.5 μL (10–100 ng) of the RT product, containing complementary DNA (cDNA), were submitted to real-time PCR using 10 μL TaqMan TM Fast Advanced Master Mix (Applied Biosystems) and 1 μL of assay (20X) containing sense and antisense primers and Taqman1probes (Applied Biosystems) specific to each gene: *NOS3* (NM_021838.2), *EDN1* (NM_012548.2), *VEGF* (NM_001110333.2), and *PIK3R2* (NM_022185.2) (Applied Biosystems, Foster City, United States). The quantitative analysis was performed in the detection system “Applied Biosystems StepOne Plus” using the commercial kit TaqMan qPCR (Invitrogen) according to the manufacturer’s instructions. The mRNA of the target genes was normalized by GAPDH – Glyceraldehyde 3 phosphate dehydrogenase (NM_017008.4). Reactions were performed in duplicate and gene expression was calculated using the comparative CT (critical threshold cycle) method (2-ΔΔCT) [34].

### 2.8. Reanalysis of HIIT datasets

As no datasets involving aging hypertensive humans subjected to HIIT were identified, the analysis was conducted using datasets obtained under conditions most closely aligned with our animal experimental model, involving healthy young individuals.

To validate our results, we retrieved a transcriptomic dataset (GSE109657) of 11 healthy young men who performed a 6-week HIIT program involving exhaustive 6–7 sets of 20-s cycling periods with 10-s rests (total of 22 samples, 11 pre-HIIT and 11 post-HIIT) for differential expression analysis. The authors study used the muscle biopsy samples collected from the vastus lateralis before and after the HIIT program [35]. Another transcriptomic dataset (GSE163356) was analyzed of nine healthy young men who performed nine sessions of HIIT (10 x 4 minutes of cycling at 91% of HRmax) over three weeks. Before the 1st and after the 9th exercise bout, the RNA sequencing skeletal muscle biopsies were obtained [36].

The raw data were collected, and the differential expression analysis was performed through the web tool NetworkAnalyst 3.0 (https://www.networkanalyst.ca/) using the limma-voom pipeline [37,38]. The relative expressions of *NOS3, PIK3R2,* and *VEGF* were displayed through a heatmap using the MORPHEUS tool (https://software.broadinstitute.org/morpheus/) [39]. In dataset GSE109657, the *EDN1* expression was not analyzed; thus, we only represent the *EDN2* transcriptional variant in the heatmap. In dataset GSE163356, *EDN1* expression was not available.

### 2.9. Statistical analyses

The data are presented as mean ± standard deviation or medians (interquartile range). The normality and homogeneity of the data were analyzed using the Shapiro-Wilk and Levene tests, respectively. The analyses of distance and arterial pressure were compared using two-way mixed-design ANOVAs (between-subjects factor: Group [Sedentary vs. HIIT]; within-subjects factor: Time, with Greenhouse-Geisser correction applied where sphericity could not be assumed. Bonferroni-adjusted pairwise comparisons were used to compared distance at baseline and 4–8 weeks. Gene expressions were assessed using the Mann-Whitney U test and effect sizes are reported as rank-biserial correlation (r) with 95% confidence intervals. The p-values were adjusted for multiple comparisons using the Benjamini-Hochberg false discovery rate (FDR) procedure. Effect sizes and their respective 95% confidence intervals were added. The significance level was established as *p* < 0.05. Statistical analyses were performed using R (version 4.6.1) in RStudio IDE (version 2026.8.1.195; Posit Software, PBC, Boston, MA, USA).

## 3. Results

### 3.1. Effects of HIIT on physical performance

Maximal treadmill velocity testing was used to determine the exercise capacity of the rats. For exercise capacity, this revealed significant main effects of HIIT (F(1,18)= 57.78, p < 0.001, η²p = 0.76, 95% CI [0.57, 1.00]) and time (F(1.78, 32.12)= 26.96, *p* < 0.001, η²p = 0.60, 95% CI [0.40, 1.00]). There was a significant Group × Time interaction (F(1.78, 32.12) = 69.68, *p* < 0.001, η²p = 0.80, 95% CI [0.68, 1.00]). No significant difference between groups at baseline (*p* = 0.53), but significant divergence by Week 4 (*p* < 0.0001) and Week 8 (*p* < 0.0001), with the HIIT group showing greater exercise capacity (Fig 3).

**Fig 3:**
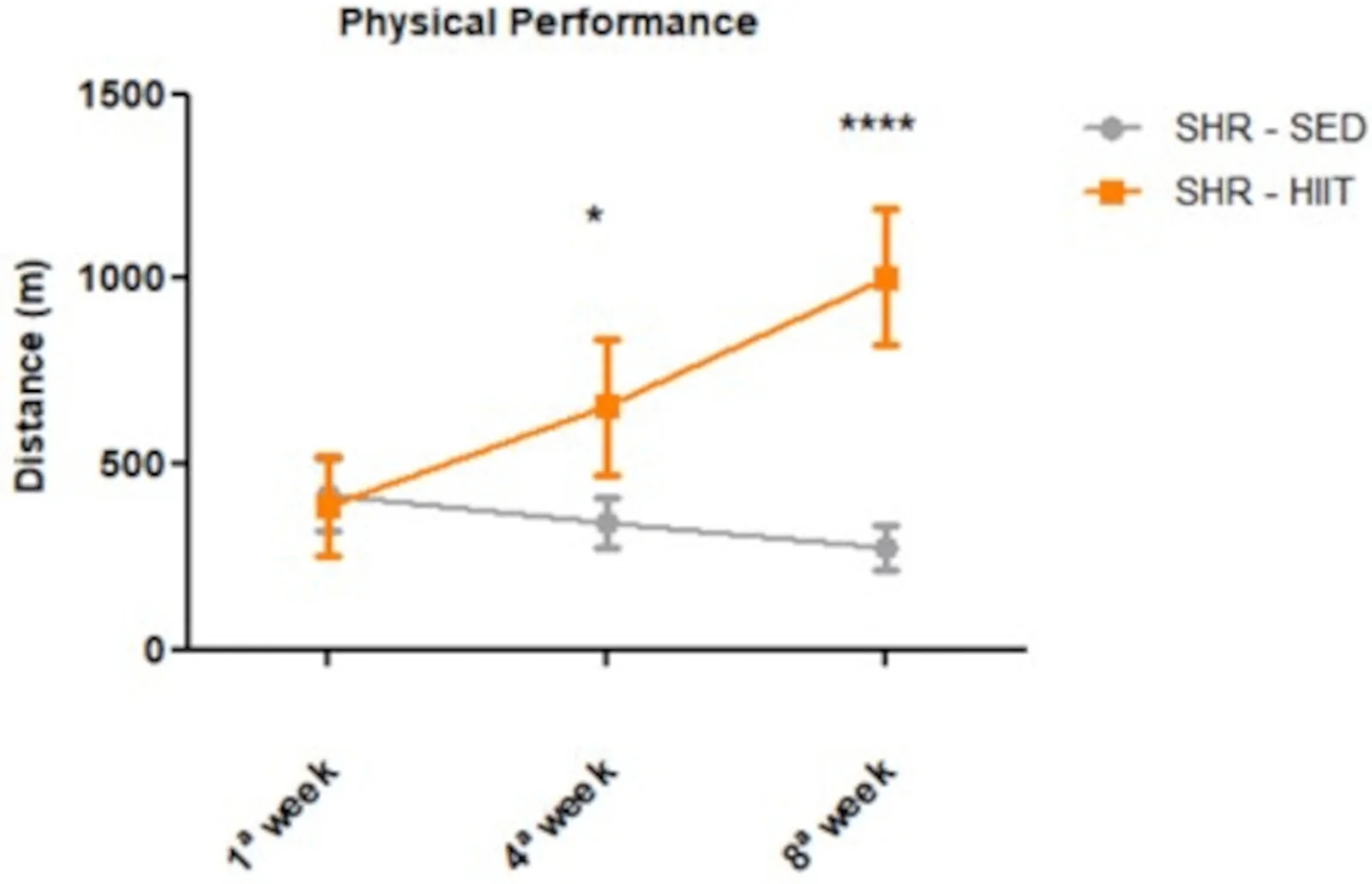
Maximum exercise capacity. Distance in the incremental treadmill test for sedentary SHR (SHR-SED) and HIIT-trained SHR (SHR-HIIT). Two-way mixed-design ANOVA. Mean ± SD. ****p < 0.0001 vs. SHR-SED.

### 3.2. HIIT improved systolic blood pressure

For systolic blood pressure, we found a significant main effect of group (F(1,18) = 8.68, *p* = 0.009, η²p = 0.33, 95% CI [0.06, 1.00]), no significant main effect of time (F(1,18) = 0.01, *p* = 0.913, η²p < 0.001, 95% CI [0.00, 1.00]), and a significant group × time interaction (F(1,18) = 10.43, *p* = 0.005, η²*p* = 0.37, 95% CI [0.09, 1.00]). Groups did not differ at baseline (*p* = 0.44) but differed significantly by Week 8 (p < 0.0001), with the HIIT group showing lower systolic blood pressure (Fig 4).

**Fig 4:**
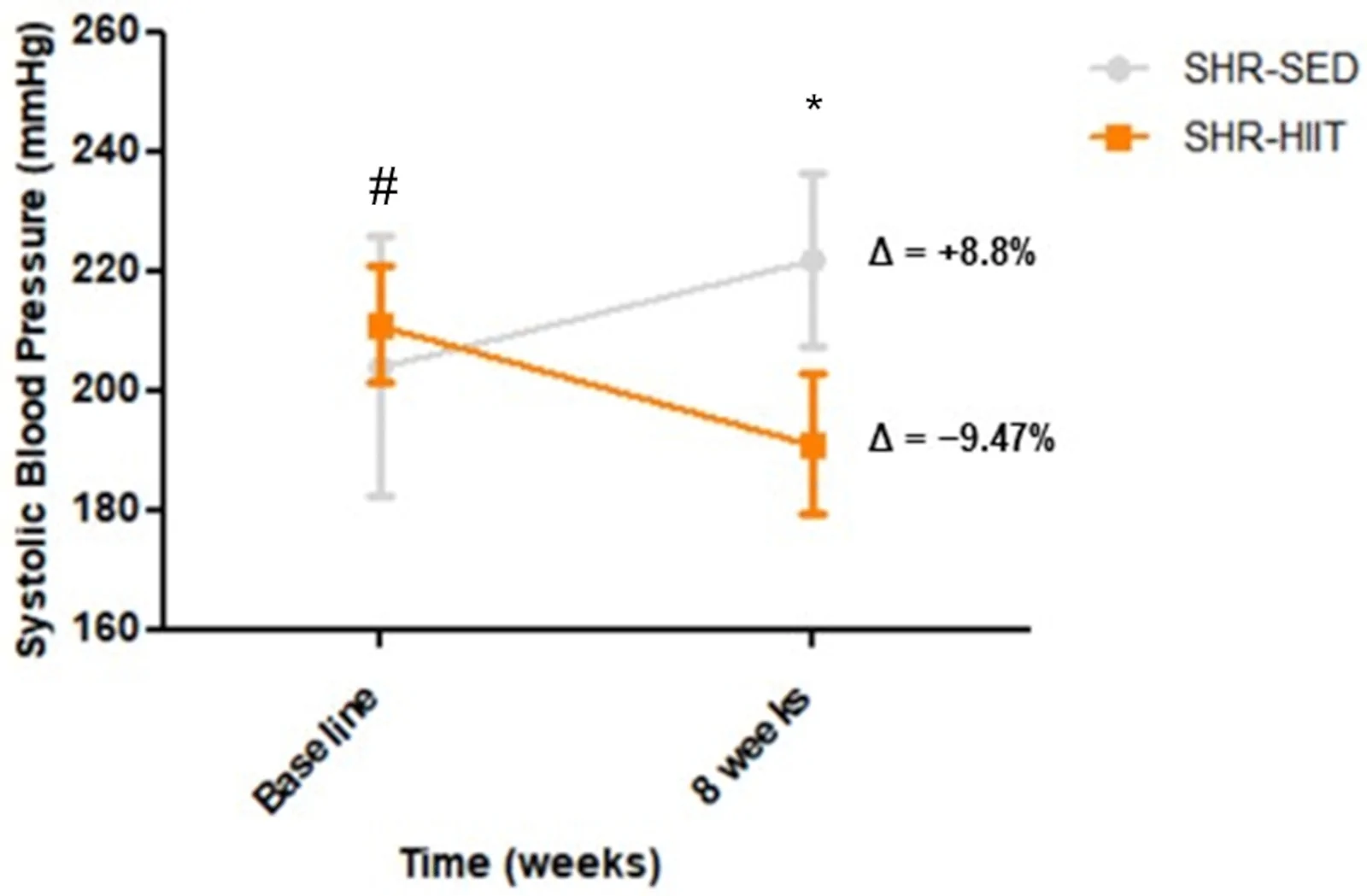
Systolic blood pressure. Mean ± SD (n = 10/group) with percentage change (∆).Two-way mixed-design ANOVAs. # *p* = 0.005 interaction group×time and *p < 0.001 vs. SHR-SED.

### 3.3. HIIT did not affect the expression of genes involved in endothelial function and vasomotor pattern in skeletal striated musculature

To further investigate the effects of HIIT on genes involved in endothelial function and vasomotor regulation, we analyzed the expression of *NOS3, EDN1, PIK3R2*, and *VEGF* in different muscle types from sedentary and trained rats. No significant differences were observed between SHR-SED and SHR-HIIT groups for any of the gene expression markers assessed in soleus or diaphragm muscle (NOS3, EDN1, VEGF, PIK3R2; all p ≥ 0.089, FDR-adjusted p ≥ 0.31). In addition, although substantial variability was detected in *EDN1* expression in the soleus muscle, this did not reach statistical significance (Fig 5). Effect sizes (rank-biserial correlation) and their respective 95% confidence intervals for all comparisons are reported in Table 1.

**Table 1:** Group comparisons (SHR-SED vs. SHR-HIIT) for gene expression markers in soleus and diaphragm muscle.

Muscle	Gene	n (SED/HIIT)	U	p	p (FDR)	r [95% CI]
Soleus	NOS3	10/10	73	0.089	0.313	0.46 [−0.02, 0.77]
Soleus	EDN1	10/9	39	0.661	0.661	−0.13 [−0.58, 0.38]
Soleus	VEGF	9/10	57	0.356	0.475	0.27 [−0.25, 0.67]
Soleus	PIK3R2	10/10	67	0.218	0.348	0.34 [−0.16, 0.70]
Diaphragm	NOS3	9/9	58	0.136	0.313	0.43 [−0.09, 0.77]
Diaphragm	EDN1	10/10	30	0.138	0.313	−0.40 [−0.74, 0.09]
Diaphragm	VEGF	9/10	27	0.156	0.313	−0.40 [−0.74, 0.11]
Diaphragm	PIK3R2	10/10	43	0.631	0.661	−0.14 [−0.58, 0.36]

Mann-Whitney U test; effect sizes are reported as rank-biserial correlation (r) with 95% confidence intervals. p-values were adjusted for multiple comparisons using the Benjamini-Hochberg false discovery rate (FDR) procedure.

**Fig 5:**
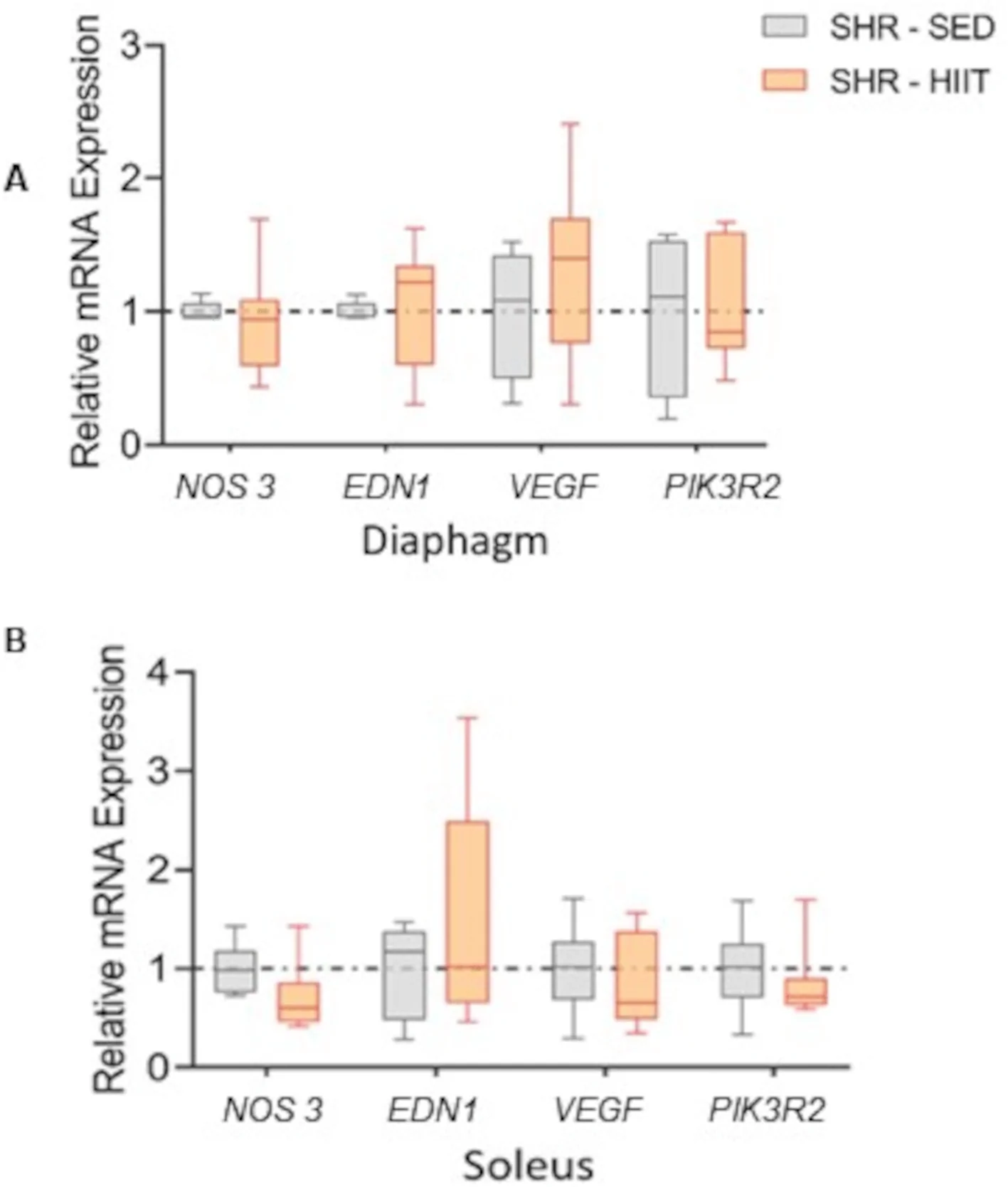
HIIT did not alter the expression levels of *NOS3, EDN1, VEGF,* and *PIK3R2.* mRNA levels of *NOS3, EDN1, VEGF,* and *PIK3R2* in the diaphragm (A) and soleus muscles (B) of sedentary and trained animals. Statistical significance was analyzed by the Mann-Whitney U test.

To validate our findings, we investigated the expression of these genes in samples from healthy young men who underwent 6 weeks of HIIT (GSE109657). Muscle biopsy samples were collected from the vastus lateralis before (PRE) and after (POST) the 6-week HIIT protocol [35]. Our reanalysis showed no differences between the genes of interest, corroborating the findings regarding the non-alteration of gene expression after HIIT (Fig 6).

**Fig 6:**
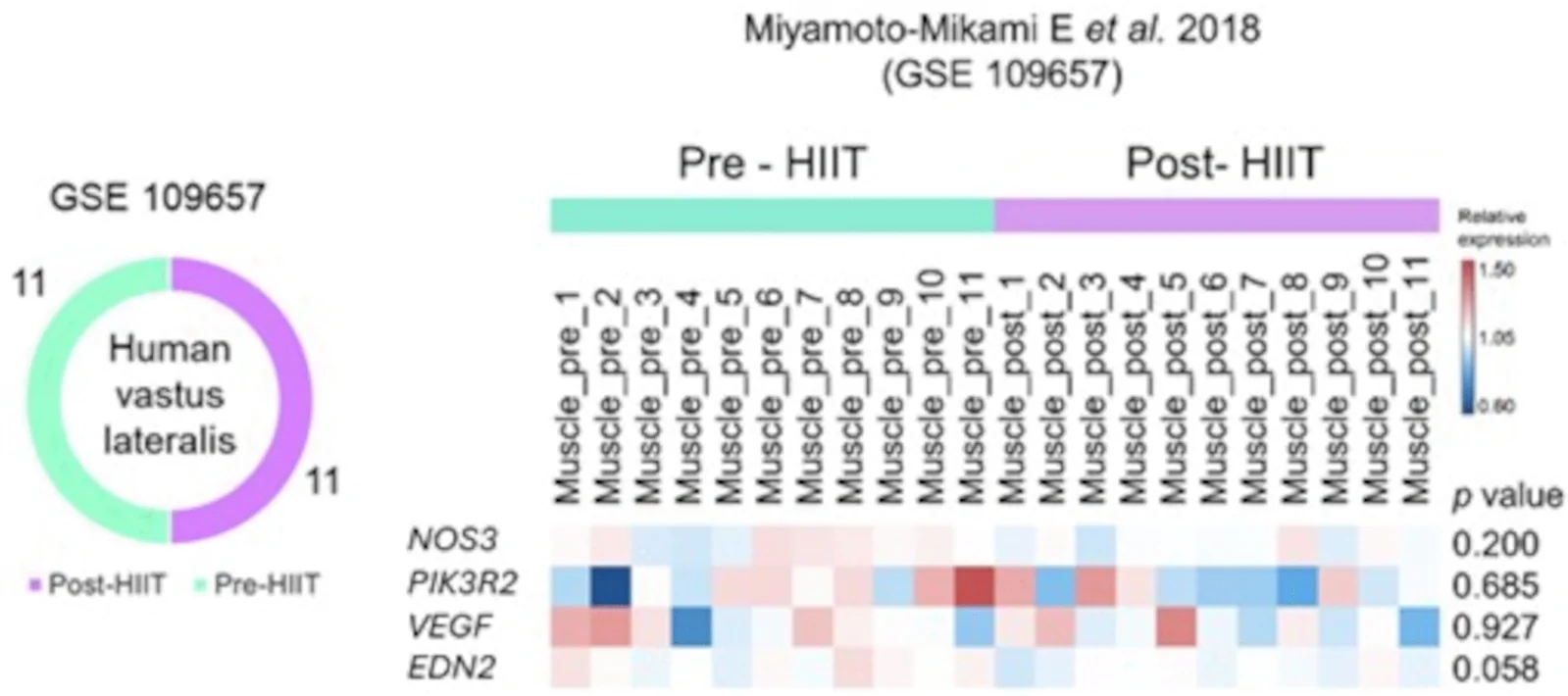
Reanalysis of *NOS3*, *PIK3R2,* and *VEGF* expression in humans submitted to HIIT. Reanalysis of the GSE109657 dataset, comparing the expression in vastus lateralis muscles from 11 subjects PRE-HIIT and 11 subjects POST-HIIT. On the right, an unsupervised hierarchical clustering heatmap presents the normalized gene expression of *NOS3*, *PIK3R2*, *EDN2,* and *VEGF* in each sample. In this dataset, the *EDN1* expression was not analyzed; thus we only represent the *EDN2* transcriptional variant in the heatmap.

These genes were analyzed in samples from healthy young men who underwent 3 weeks of HIIT (GSE163356). Muscle biopsy samples were collected from the vastus lateralis before (PRE) and after (POST) the 3-week HIIT [36]. Our reanalysis showed differences between the genes of interest (Fig 7).

**Fig 7:**
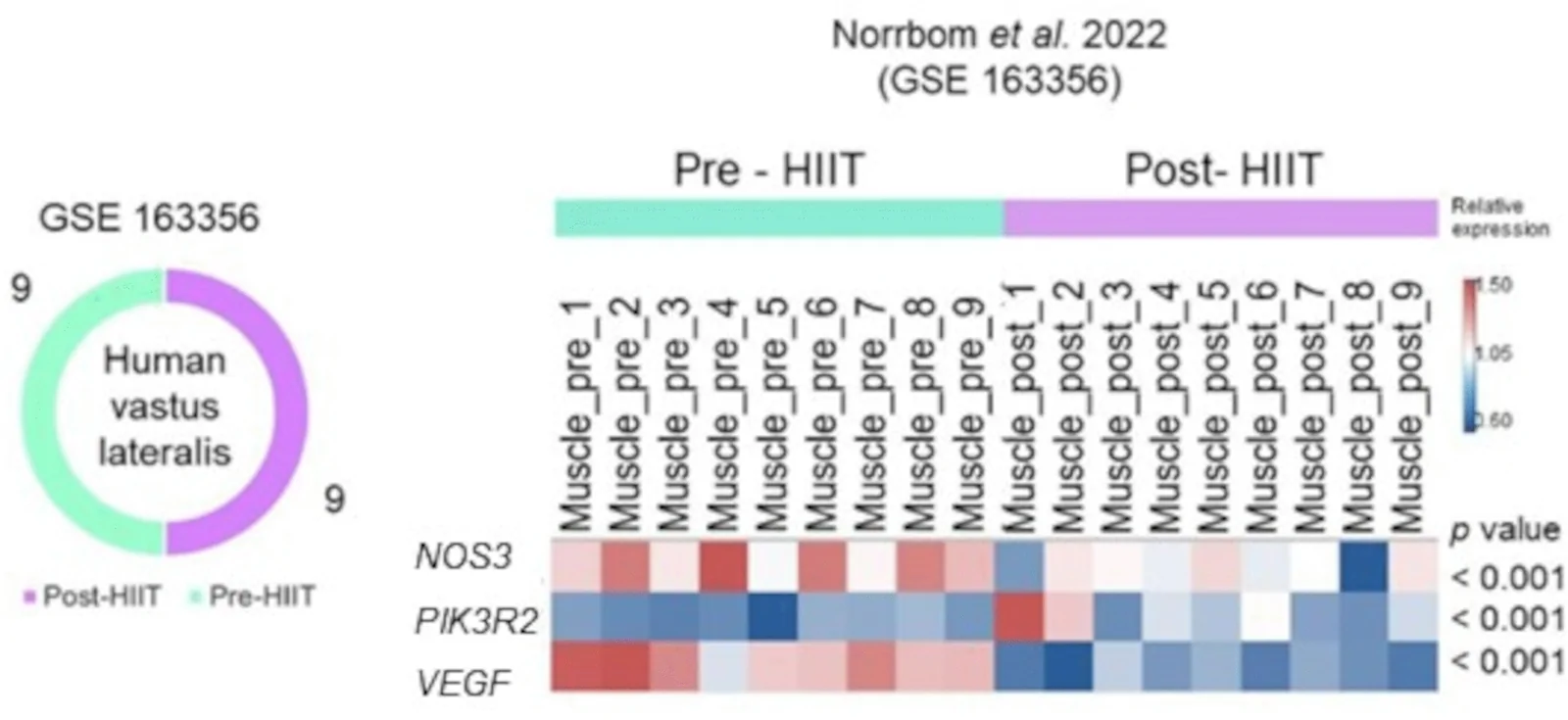
Reanalysis of *NOS3*, *PIK3R2,* and *VEGF* expression in humans submitted to HIIT. Reanalysis of the dataset GSE163356, comparing the gene expression in vastus lateralis muscles from 9 subjects PRE-HIIT and 9 subjects POST-HIIT. On the right, an unsupervised hierarchical clustering heatmap presents the normalized gene expression of *NOS3*, *PIK3R,2* and *VEGF* in each sample. *EDN1* expression was not available in this dataset.

Fig 8 summarizes the main findings of the present study and illustrates the potential mechanisms underlying the observed physiological adaptations following HIIT.

**Fig 8:**
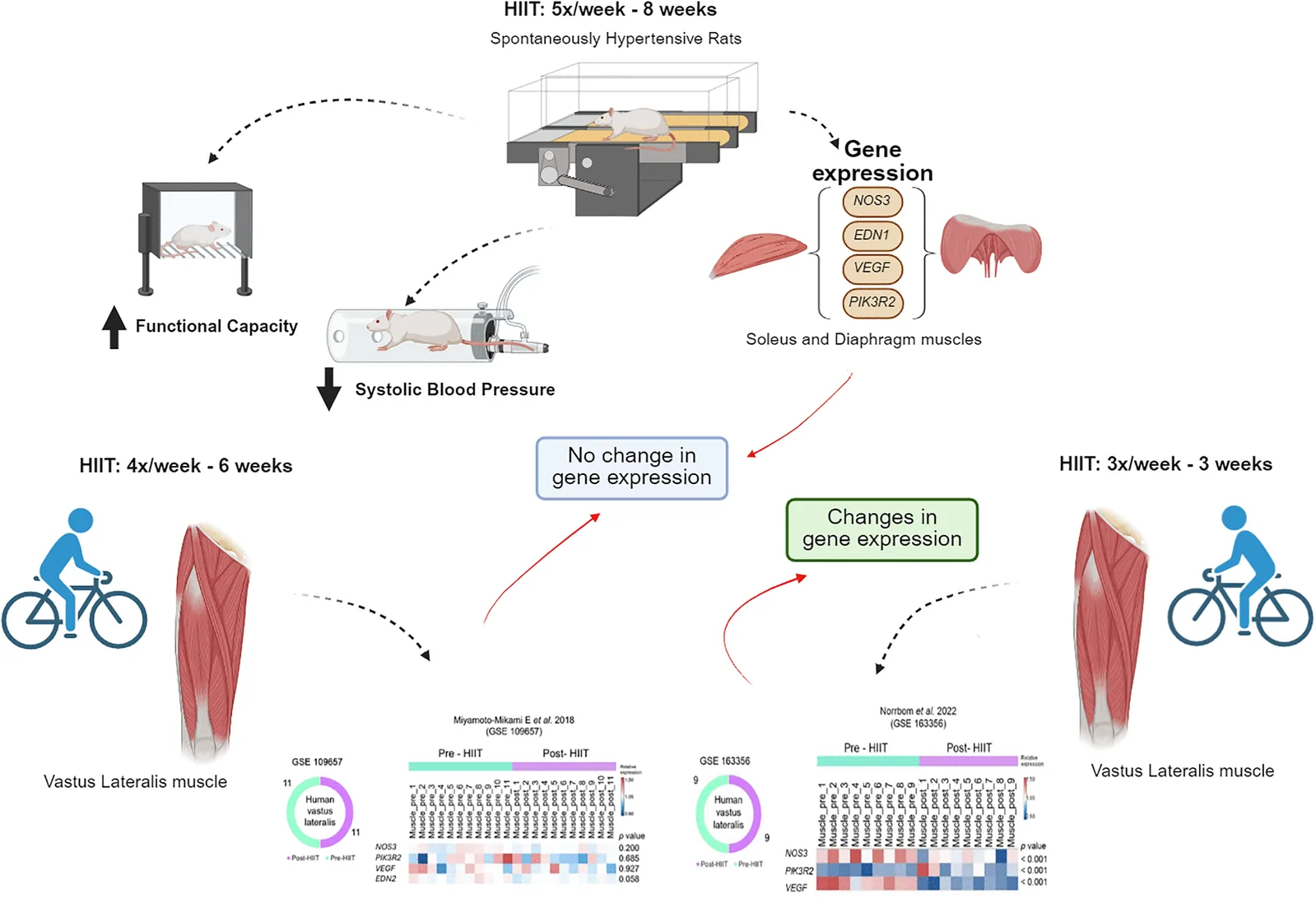
Schematic representation of comparisons of changes in gene expression among the experimental results and reanalysis of two HIIT datasets.

## 4. Discussion

Hypertension is a chronic condition and a major global driver of cardiovascular disease [2,40]. There is evidence that HIIT could be prescribed as a feasible and time-efficient option for clinical interventions for individuals with hypertension, to improve VO_2_peak, blood pressure, and resting heart rate [20,41]. In the current study, we assessed the influence of HIIT on the gene expression of vasomotor and angiogenesis markers (*NOS3, VEGF, EDN1,* and *PIK3R2*) in two metabolically distinct skeletal muscles – the soleus and diaphragm muscles – of aging SHR. Considering that skeletal muscle comprises up to half of the lean body mass, which makes muscle tissue an important determinant of total peripheral resistance [14], and that skeletal muscle adaptations are essential to bring about improvements in functional capacity induced by physical exercise, the present manuscript aimed to evaluate the expression of genes encoding proteins involved in vasomotor regulation and angiogenesis in skeletal muscle. The diaphragm was deliberately selected because it is indispensable to effective ventilation and respiratory homeostasis, while the soleus muscle was chosen due to its critical contribution to efficient gait and its essential role in postural maintenance and antigravity function. Our results showed that an 8-week HIIT protocol might promote decreased arterial pressure and improved physical condition without affecting the gene expressions of *NOS3, PIK3R2, VEGF,* and *EDN1* in soleus and diaphragm muscles (Fig 8). As in the reanalyzed datasets, the primary aim of our manuscript was to determine whether the improved physical performance observed in the evaluated Spontaneously Hypertensive Rats was associated with differential expression of *NOS3*, *PIK3R2*, *VEGF*, and *EDN1* in muscle tissue.

The SHR model adopted is widely used to study hypertension. HIIT promoted attenuation of arterial hypertension in aging SHR, which is particularly relevant since elevated arterial pressure is a strong predictor of cardiovascular morbidity and mortality [42–45]. In the present study, eight weeks HIIT promoted a attenuation of arterial hypertension, likely reflecting the duration of training required to activate mechanisms responsible for reducing blood pressure [19,26,46]. Potential contributors to the observed decrease in arterial pressure include positive modulation of the autonomic nervous system, improved baroreflex sensitivity, reduced peripheral vascular resistance, and enhanced skeletal muscle neovascularization, which contributes to reducing vascular overload [47,48]. Clinical studies support the cardiovascular benefits of HIIT. For example, Conraads et al. (2015) [49] reported that a 12-week HIIT program improved cardiorespiratory fitness, endothelial responsiveness, and perceived quality of life among patients engaged in cardiac rehabilitation. More recently, randomized trials and meta-analyses reinforced these outcomes, indicating that HIIT can elicit superior improvements in aerobic performance and vascular health when compared with moderate continuous training in individuals with cardiovascular disease [50,51]. The efficacy of high-intensity interval training (HIIT) in mitigating blood pressure levels has also been reported in rat experimental models of hypertension. The implementation of HIIT protocols has been shown to significantly reduce systolic blood pressure in both young and aged SHR [26,44,52–56]. In addition to lowering blood pressure, HIIT promoted a substantial increase in functional capacity and exercise tolerance, as well as attenuated the effects caused by systemic arterial hypertension in organs such as urinary bladder [54] and prostate [56]. In line with these findings, our results revealed an 82.7% increase in the running distance achieved by the SHR-HIIT group after eight weeks of training, indicating a substantial improvement in exercise capacity.

In this study, the gene expression of *NOS3* and *EDN1* in the soleus and diaphragm muscles remained unchanged after 8 weeks of HIIT. Moderate-intensity physical exercise in hypertension generates molecular effects, including alterations in vasoactive markers, mainly an increase endothelial nitric oxide synthase (eNOS) and NO [23,57], and a decrease in *EDN1* when assessing in blood samples levels [58,59]. In the tibialis anterior, 8 weeks of aerobic training increased vessel density and eNOS protein level [57].Nyberg, Mortensen, and Hellsten (2013) [16] reported that skeletal muscle and plasma endothelin-1 levels increase with age, and demonstrated that aerobic exercise training counteracts the age-related elevation of endothelin-1 in the vastus lateralis and in plasma, and normalizes plasma endothelin-1 levels in individuals with essential hypertension. This mechanism may contribute to the cardiovascular advantages associated with exercise training in older and hypertensive populations. Richardson et al. (1999) [60] reported an increase in *VEGF* mRNA following acute exercise, supporting its involvement in exercise-induced skeletal muscle angiogenesis. In contrast, the current study, which assessed gene expression after eight weeks of intensity training, did not detect significant changes. This finding is consistent with the systematic review and meta-regression by Mølmen Almquist and Skattebo (2025) [61], which concluded that increases in capillarization occur primarily during the early stages of HIIT, typically within the first four weeks. Importantly, the absence of changes in mRNA expression does not necessarily indicate the absence of biological adaptation. Physiological adaptations to exercise may occur through post-transcriptional, translational, and post-translational regulatory mechanisms that are not fully captured by gene expression analysis alone [62,63]. Furthermore, variations in tissue type may explain the observed discrepancy between the present findings and those reported in previous studies. In a systematic review [23], NO and ET-1 levels were increased by intensity exercise, but the assessment methods used were different (serum, plasma, vascular function). In addition, the different results may also be related to the stage of hypertension (compensated phase) and to the characteristics of the analyzed muscles, which are predominantly oxidative and composed of slow-contracting fibers [59,64,65]..

In the current study, *VEGF* and *PIK3R2* expression in the soleus and diaphragm muscles were similar across groups, which may reflect the temporal dynamics of these genes following prolonged exercise stimuli. *VEGF* expression typically increases during the early stages of physical training but tends to return to baseline once capillary density increases [8,66,67]. This pattern is consistent with the dynamic regulation of angiogenic signaling during exercise-induced vascular remodeling, in which *VEGF* plays a central role in mediating capillary growth and vascular adaptation to increased metabolic and hemodynamic demands [68,69]. Exercise training is known to stimulate angiogenic processes in skeletal muscle through multiple mechanisms, including mechanical stimuli, shear stress, and metabolic signals that regulate endothelial cell function and capillary formation [68,69]. Consequently, transient increases in *VEGF* expression may occur during the early phases of training when angiogenesis is actively being initiated, whereas gene expression may normalize once structural vascular adaptations are established. This response may also involve post-transcriptional regulatory mechanisms, including microRNAs such as miR-126, which has been associated with angiogenic signaling and modulation of *VEGF*-related pathways. In hypertension, miR-126 expression has been reported to be reduced in skeletal muscle but may increase following exercise training [70,71].

More broadly, exercise-induced adaptations involve complex molecular regulation that occurs at multiple biological levels, including gene transcription, protein synthesis, enzymatic activity, and intracellular signaling pathways [62,63,72]. Therefore, the absence of detectable changes in the expression of angiogenesis-related genes after prolonged training may reflect the dynamic and time-dependent regulation of these pathways rather than the absence of physiological adaptation. In this context, once vascular remodeling and capillary expansion have occurred, cellular homeostasis may restore the expression of angiogenesis-related genes toward basal levels despite the persistence of functional vascular adaptations.

Because transcriptomic data from hypertensive humans undergoing HIIT are scarce, we analyzed datasets from healthy individuals. In GSE163356, short-term HIIT altered angiogenic and vasomotor genes in the vastus lateralis [36]. However, after six weeks, no significant changes were observed [35], highlighting the time-dependent nature of HIIT adaptations in skeletal muscle.

Some limitations of our study should be considered, for example, the evaluation of skeletal muscle gene expression alone does not allow direct conclusions regarding vascular functional adaptations or protein-level regulation. Exercise adaptations involve complex molecular networks that extend beyond transcriptional regulation, including translational and post-translational mechanisms [62,63]. Additionally, it is important to emphasize the relevance of conducting such investigations by also examining the aorta, a major blood vessel that is directly affected by systemic arterial hypertension.

The consistency of the findings reported herein, with transcriptomic data from human studies reinforces the translational relevance of this model and indicates that improvements in cardiovascular and functional outcomes following HIIT are not necessarily accompanied by detectable changes in skeletal muscle endothelial gene expression. The lack of transcriptional alterations does not rule out the occurrence of functional adaptations to skeletal muscles, mediated by post-transcriptional, translational, or post-translational processes. These findings suggest that long-term HIIT adaptations may not be mirrored by changes in skeletal muscle gene expression, and that complementary vascular or protein-level analyses are necessary to fully clarify the mechanisms driving the observed reduction in blood pressure.

In conclusion, eight weeks of high-intensity interval training promoted an attenuation of arterial hypertension and improved exercise capacity in hypertensive rats, without altering *NOS3, EDN1, PIK3R*2, or *VEGF* expression in the soleus and diaphragm muscles.
