# Supplementary material for: Endothelial-related gene expression profile of muscle adaptation to high-intensity interval training in arterial hypertension
Source: PLoS One. 2026 Oct 14;21(10):e0358696. doi: 10.1371/journal.pone.0358696 (PMC13667785; doi:10.1371/journal.pone.0358696)
Supplement: Supplementary file 1 [file pone.0358696.s002.docx]

***Dra Francis Lopes Pacagnelli***

***University of Western São Paulo - UNOESTE | Brazil***

*Dear Emily Chenette*

*Editor-in-Chief*

*Plos One*

*April 30, 2026.*

Thank you for the opportunity to submit a revised version of our manuscript. We appreciate the time and effort that you have dedicated to providing your valuable feedback on our work. We are grateful to the reviewers for their insightful comments. We have been able to incorporate changes to reflect most of the suggestions provided by the reviewers and have highlighted the changes in the revised manuscript.

Here is a point-by-point response to the reviewers’ comments and concerns.

We look forward to hearing from you in due course.

Yours sincerely,

Dra Francis Lopes Pacagnelli

**Reviewer #1:**

Thank you for the opportunity to revise our manuscript. We are grateful for your careful and considered comments. We have made every attempt to respond to each (see below) and to fully address your comments in the revised manuscript. We believe these revisions have resulted in a significantly improved manuscript.

**Major**

1- **The authors report that HIIT improved blood pressure and exercise capacity without altering the expression of NOS3, VEGF, EDN1, or PIK3R2. Given that these measurements were obtained after a prolonged training period, the lack of transcriptional differences suggests that chronic HIIT did not induce sustained remodeling of these pathways in the tissues examined. However, the absence of transcriptional changes does not preclude functional adaptation occurring through post-transcriptional, translational, or post-translational mechanisms. This distinction should be more clearly stated, as the current version may imply that no biologically meaningful adaptation occurred. I would suggest the authors expand their discussion by suggesting that chronic adaptations may not be reflected in skeletal muscle gene expression and that vascular or protein-level analyses would be required to fully elucidate the mechanisms underlying the observed blood pressure reduction.**

**RESPONSE:** We thank the reviewer for this important and constructive comment. We agree that the absence of changes in mRNA expression does not necessarily indicate the absence of biological adaptation. In the revised manuscript, we have expanded the Discussion to clarify that exercise-induced physiological responses may occur through post-transcriptional, translational, or post-translational regulatory mechanisms that are not fully captured by gene expression analysis alone. **(Discussion: Pages 17-18, Lines 413-439)**

2- **Line 352. When comparing the present findings with previous studies reporting increased NOS3 expression or reduced EDN1 levels following moderate-intensity exercise (e.g., refs 46–48), the authors should acknowledge that those studies primarily assessed vascular tissues or endothelial function, rather than skeletal muscle gene expression. In contrast, the present study evaluated gene expression in soleus and diaphragm muscles. Because tissue specificity may influence transcriptional responses, the comparison between vascular-based studies and skeletal muscle mRNA measurements should be interpreted with caution. Therefore,** **the differences in tissue type may account for the apparent discrepancy between the current findings and prior literature, rather than implying that training intensity alone explains the divergent results.**

**RESPONSE:** We have also clarified that molecular responses to exercise may vary across tissues and biological levels of regulation. Therefore, we agree that comparisons between studies evaluating vascular function and those assessing gene expression in skeletal muscle should be interpreted with caution. **(Introduction: Page 5, Lines 119-124; and Discussion: Page 17, Line 418-420).**

We have indicated this aspect as a limitation of the study, highlighting that future investigations including vascular functional analyses or protein-level measurements are necessary to better elucidate the mechanisms underlying the observed reduction in blood pressure following HIIT. **(Discussion: Page 19, Line 453- 459)**

**Minor**

3- **Line 240. Please clarify which statistical test was used (e.g., one-way ANOVA or two-way ANOVA). The Statistical Analysis section refers to Tukey’s test, whereas the figure legend mentions Bonferroni. Please ensure consistency and specify which post hoc test was applied. Please specify the version of the statistical software used for analyses. Please clarify the post hoc test used to identify differences after the Kruskal-Wallis test, or remove this information if no nonparametric test was used.**

**RESPONSE:** We have clarified the statistical tests used and reformulated this description. We apologize for this unfortunate mistake. The tests have also been included in the figure legends. The version of the statistical software used has been added. **(Page 11, Line 272)**

4- **Lines 332-333. Figure 8 appears to be discussed within the Discussion section; please consider relocating this figure to the Results section or adjusting the text accordingly.**

**RESPONSE:** We appreciate the suggestion and have relocated Fig 8 to the end of the Results section. **(Page 14, Line 346)**

**Reviewer #2:**

Thank you for the opportunity to revise our manuscript. We are grateful for your careful and considered comments. We have made every attempt to respond to each (see below) and to fully address your comments in the revised manuscript. We believe these revisions have resulted in a significantly improved manuscript.

1- **The present study investigated the impact of high-intensity interval training (HIIT) on blood pressure and endothelial-related gene expression. Overall, the Introduction section requires better organization and contextualization. The discussion of NOS3, VEGF, EDN1, and related markers appears disconnected and should be better integrated to provide a clearer rationale. Additionally, the authors should explain more explicitly how high-intensity exercise may influence gene expression, particularly the genes investigated in the present study.**

**RESPONSE:** We thank the reviewer for this important and constructive comment. The Introduction section has been reorganized to improve contextualization. In addition, the discussion of *NOS3, VEGF, EDN1*, and related markers has been rewritten to provide a clearer rationale and we have added explanations of how high-intensity exercise can influence the expression of the genes under investigation**. (Pages 3-5, Lines 76-108; 118-131)**

2- **The Discussion section is somewhat speculative and would benefit from the inclusion of additional studies examining the effects of HIIT on blood pressure responses in animal models, particularly in rats.**

**RESPONSE:** We appreciate the suggestion. Additional studies showing the effects of HIIT on blood pressure responses in rats have been included. **(Page 16, Line 388-394)**

**Specific comments:**

3- **Lines 74–80: Please clarify whether the reported changes in gene expression are directly related to hypertension and, if so, explain the underlying rationale more clearly.**

**RESPONSE:** We hope that this information strengthens the text and helps clarify the relationship between the investigated genes and arterial hypertension. **(Page 3-5, Lines 74-108)**

4- **Line 85: The phrase “loss of functional integrity” is unclear. Please specify which tissue or structure is being referred to.**

**RESPONSE:** We thank the reviewer for this suggestion and have rewritten the relationships between alterations in EDN1 gene expression and their effects on endothelial dysfunction. **(Page 4, Lines 96-102)**

5- **Line 86: Is the association between increased EDN1 and endothelial dysfunction explained solely by its regulation within the central nervous system? Please revise and clarify this statement.**

**RESPONSE:** We thank the reviewer for this suggestion and have revised the sentence to clarify that the association between EDN1 and endothelial dysfunction is not restricted to regulation within the central nervous system. The revised text now also emphasizes that endothelin-1 is a potent vasoconstrictor peptide produced by endothelial cells and involved in the regulation of vascular tone and peripheral vascular resistance. **(Page 4, Lines 96-102)**

6- **Lines 101–113: It is recommended to include appropriate references to support the statements made in this paragraph.**

**RESPONSE:** We have included more references to support the statements**. (Page 5, Lines 115-124)**

7- **Lines 106–107: The background information presented does not adequately support the study’s hypothesis. This section should be revised to ensure a clearer logical connection between background evidence and the proposed hypothesis.**

**RESPONSE:** We thank the reviewer for this valuable suggestion. The Introduction section has been revised to improve the logical connection between the background information and the study hypothesis. Additional references have also been included to support the role of exercise in molecular signaling and angiogenic adaptations relevant to vascular function and blood pressure. **(Page 5, Lines 118-131)**

8- **Lines 169–170: Please briefly describe how the exercise protocol was performed to improve clarity in the Methods section.**

**RESPONSE:** The exercise protocol performed has been described. (**Page 8, Lines 195-199)**

9- **Line 244: Please confirm whether a two-way ANOVA was used and clarify the factors included in the analysis.**

**RESPONSE:** We have clarified the statistical test used and reformulated this description. We apologize for this unfortunate mistake. The tests have also been included in the figure legends. The version of the statistical software used has been added. **(Page 11, Lines 272-275)**

10- **Lines 322–325: Should the tissue investigated be an aortic segment rather than skeletal muscle? Please clarify and revise accordingly.**

**RESPONSE:** We acknowledge the reviewer’s concern. Evaluating the aorta is highly relevant in systemic arterial hypertension, and we will address this aspect as a future direction of the present study. We have presented a better justification for choosing skeletal muscle in the discussion and pointed out the study of the aorta as a perspective. **(Page 15, Lines 360-364 and Page 19, Line 457)**

11- **Lines 328–330: Could this phenomenon be explained by a dissociation between vascular responses (e.g., reduced blood pressure) and the gene expression measured in muscle tissue? If so, this should be acknowledged as a limitation of the study.**

**RESPONSE:** We appreciate the reviewer's concern and have adjusted the discussion accordingly, pointing out the limitations of the study. Considering that gene expression measurements were obtained after a prolonged training period, the absence of transcriptional differences suggests that chronic HIIT did not induce sustained remodeling of these pathways in the examined tissues. However, the absence of transcriptional changes does not exclude the possibility of functional adaptation through post-transcriptional, translational, or post-translational mechanisms. We have explained this more clearly in the discussion so as not to imply that no biologically significant adaptation occurred. We have also expanded the discussion, suggesting that chronic adaptations in blood pressure and functional capacity were not reflected in skeletal muscle gene expression and that vascular or protein-level analyses would be necessary to fully elucidate the mechanisms underlying the observed reduction in blood pressure. **(Page 17, Lines 413-423 and Page 19, Lines 453-459)**

12- **Line 333: Please remove the statement “Image created in BioRender.com.”**

**RESPONSE:** The references to the use of BioRender in Figures 1 and 8 have been removed, as suggested, to maintain conciseness and avoid unnecessary information in the figure legends. **(Page 15, Line 349)**

The reviewers’ observations were certainly crucial for strengthening the robustness of this manuscript, and we hope that we have adequately addressed all of their suggestions.
