# Supplementary material for: Endothelial-related gene expression profile of muscle adaptation to high-intensity interval training in arterial hypertension
Source: PLoS One. 2026 Oct 14;21(10):e0358696. doi: 10.1371/journal.pone.0358696 (PMC13667785; doi:10.1371/journal.pone.0358696)
Supplement: Supplementary file 2 [file pone.0358696.s003.docx]

**Response to Reviewers**

Thank you for the opportunity to revise our manuscript. We are grateful for your careful and considered comments. We have made every attempt to respond to each (see below) and to fully address your comments in the revised manuscript. We believe these revisions have resulted in a significantly improved manuscript

**Reviewer #1:**

*“In the statistical analysis section of the revised manuscript, the authors stated that distance and blood pressure were compared using t-tests. This approach is not appropriate for these outcomes because exercise capacity and systolic blood pressure were measured repeatedly over time. Exercise capacity was assessed at baseline, week 4, and week 8, while systolic blood pressure was assessed at baseline and week 8. Therefore, the analysis should account for both group and time effects”.*

**Response:** We thank the reviewer for this observation and agree that t-tests were not appropriate given the repeated-measures structure of these outcomes. We have re-analyzed both variables using two-way mixed-design ANOVAs (between-subjects factor: Group [Sedentary vs. HIIT]; within-subjects factor: Time), with Greenhouse-Geisser correction applied where sphericity could not be assumed.

**The results were altered:**

For exercise capacity, this revealed significant main effects of Group (F(1,18) = 57.78, p < .001, η²p = .76, 95% CI [.57, 1.00]) and Time (F(1.78, 32.12) = 26.96, p < .001, η²p = .60, 95% CI [.40, 1.00]), and a significant Group × Time interaction (F(1.78, 32.12) = 69.68, p < .001, η²p = .80, 95% CI [.68, 1.00]). Bonferroni-adjusted pairwise comparisons showed no significant difference between groups at baseline (p = .534), but significant divergence by Week 4 (p < .0001) and Week 8 (p < .0001), with the HIIT group showing greater exercise capacity.

For systolic blood pressure, we found a significant main effect of Group (F(1,18) = 8.68, p = .009, η²p = .33, 95% CI [.06, 1.00]), no significant main effect of Time (F(1,18) = 0.01, p = .913, η²p < .001, 95% CI [.00, 1.00]), and a significant Group × Time interaction (F(1,18) = 10.43, p = .005, η²p = .37, 95% CI [.09, 1.00]). Groups did not differ at baseline (p = .448) but differed significantly by Week 8 (p < .0001), with the HIIT group showing lower systolic blood pressure. The Statistical Analysis, Results, and Table X/Y of the revised manuscript have been updated accordingly.

**Reviewer #2:**

*"* *I recommend reporting effect sizes for the main statistical analyses, as they provide important information regarding the practical significance of the findings beyond statistical significance alone. In addition, please report the corresponding 95% confidence intervals for the effect sizes."*

**Response:** We agree and have added effect sizes with 95% confidence intervals throughout. For the ANOVA main and interaction effects, we report partial eta-squared (η²p) with 95% CIs (Table X). For pairwise group comparisons at each timepoint, we report Cohen's d with 95% CIs (Table Y). These results were added in text.

We have also extended this to the gene expression data (soleus and diaphragm NOS3, EDN1, VEGF, PIK3R2), reporting rank-biserial correlation (r) with 95% CIs alongside the Mann-Whitney U test results already used for these comparisons. The Table 1 was added in results.

**Table X —** **ANOVA main and interaction effects, we report partial eta-squared (η²p) with 95% Cis**

| **Outcome** | **Effect** | **df** | **F** | ***p*** | **Partial η² [95% CI]** |
| --- | --- | --- | --- | --- | --- |
| **Exercise capacity** | Group | 1.18 | 57.78 | <0.001 | 0.76 [0.57, 1.00] |
|  | Time | 1.78, 32.12 | 26.96 | <0.001 | 0.60 [0.40, 1.00] |
|  | Group × Time | 1.78, 32.12 | 69.68 | <0.001 | 0.80 [0.68, 1.00] |
| **Systolic blood pressure** | Group | 1.18 | 8.68 | 0.009 | 0.33 [0.06, 1.00] |
|  | Time | 1.18 | 0.01 | 0.913 | <0.001 [0.00, 1.00] |
|  | Group × Time | 1.18 | 10.43 | 0.005 | 0.37 [0.09, 1.00] |

Note: η²p confidence intervals are one-sided, with the upper bound fixed at 1.00, as is standard

for variance-explained effect sizes.

**Table Y. Pairwise group comparisons**

| **Outcome** | **Timepoint** | **Sedentary − HIIT** | ***t*** | **df** | ***p*** | **Cohen’s *d* [95% CI]** |
| --- | --- | --- | --- | --- | --- | --- |
| **Exercise capacity** | Baseline | 32 | 0.63 | 18 | 0.534 | 0.28 [−0.74, 1.15] |
|  | Week 4 | −312 | −5.02 | 18 | <0.0001 | −2.25 [−5.21, −1.15] |
|  | Week 8 | −731 | −12.03 | 18 | <0.0001 | −5.38 [−11.3, −4.22] |
| **Systolic blood pressure** | Baseline | −6.8 | −0.77 | 18 | 0.448 | −0.35 [−1.09, 0.68] |
|  | Week 8 | 31.1 | 6.10 | 18 | <0.0001 | 2.73 [2.04, 4.46] |

**Table 1.** Group comparisons (SHR-SED vs. SHR-HIIT) for gene expression markers in soleus and diaphragm muscle.

| **Muscle** | **Gene** | **n (SED/HIIT)** | **U** | **p** | **p (FDR)** | **r [95% CI]** |
| --- | --- | --- | --- | --- | --- | --- |
| Soleus | NOS3 | 10/10 | 73 | 0.089 | 0.313 | 0.46 [−0.02, 0.77] |
| Soleus | EDN1 | 10/9 | 39 | 0.661 | 0.661 | −0.13 [−0.58, 0.38] |
| Soleus | VEGF | 9/10 | 57 | 0.356 | 0.475 | 0.27 [−0.25, 0.67] |
| Soleus | PIK3R2 | 10/10 | 67 | 0.218 | 0.348 | 0.34 [−0.16, 0.70] |
| Diaphragm | NOS3 | 9/9 | 58 | 0.136 | 0.313 | 0.43 [−0.09, 0.77] |
| Diaphragm | EDN1 | 10/10 | 30 | 0.138 | 0.313 | −0.40 [−0.74, 0.09] |
| Diaphragm | VEGF | 9/10 | 27 | 0.156 | 0.313 | −0.40 [−0.74, 0.11] |
| Diaphragm | PIK3R2 | 10/10 | 43 | 0.631 | 0.661 | −0.14 [−0.58, 0.36] |

Mann-Whitney U test; effect sizes are reported as rank-biserial correlation (r) with 95% confidence intervals. p-values were adjusted for multiple comparisons using the Benjamini-Hochberg false discovery rate (FDR) procedure.

**Comment 3:** *"...inconsistencies in font type or formatting. Please carefully review the document..."*

**Response:** We have reviewed the manuscript in full and corrected the font and formatting inconsistencies throughout.
