# Supplementary material for: Endothelial-related gene expression profile of muscle adaptation to high-intensity interval training in arterial hypertension
Source: PLoS One. 2026 Oct 14;21(10):e0358696. doi: 10.1371/journal.pone.0358696 (PMC13667785; doi:10.1371/journal.pone.0358696)
Supplement: Peer review history

# Peer review: Decision Letter 0

**DOI:** 10.1371/journal.pone.0358696.r001

**Authors:** Juan M. Murias

25 Jan 2026

Dear Dr. Pacagnelli,

Please submit your revised manuscript by Mar 11 2026 11:59PM. If you will need more time than this to complete your revisions, please reply to this message or contact the journal office at plosone@plos.org. When you're ready to submit your revision, log on to https://www.editorialmanager.com/pone/ and select the 'Submissions Needing Revision' folder to locate your manuscript file.

- A letter that responds to each point raised by the academic editor and reviewer(s). You should upload this letter as a separate file labeled 'Response to Reviewers'.
- A marked-up copy of your manuscript that highlights changes made to the original version. You should upload this as a separate file labeled 'Revised Manuscript with Track Changes'.
- An unmarked version of your revised paper without tracked changes. You should upload this as a separate file labeled 'Manuscript'.

If you would like to make changes to your financial disclosure, please include your updated statement in your cover letter. Guidelines for resubmitting your figure files are available below the reviewer comments at the end of this letter.

If applicable, we recommend that you deposit your laboratory protocols in protocols.io to enhance the reproducibility of your results. Protocols.io assigns your protocol its own identifier (DOI) so that it can be cited independently in the future. For instructions see: https://journals.plos.org/plosone/s/submission-guidelines#loc-laboratory-protocols. Additionally, PLOS ONE offers an option for publishing peer-reviewed Lab Protocol articles, which describe protocols hosted on protocols.io. Read more information on sharing protocols at https://plos.org/protocols?utm_medium=editorial-email&utm_source=authorletters&utm_campaign=protocols.

We look forward to receiving your revised manuscript.

Kind regards,

Juan M. Murias

Academic Editor

PLOS One

Journal Requirements:

When submitting your revision, we need you to address these additional requirements.

1. Please ensure that your manuscript meets PLOS ONE's style requirements, including those for file naming. The PLOS ONE style templates can be found at

https://journals.plos.org/plosone/s/file?id=wjVg/PLOSOne_formatting_sample_main_body.pdf and

https://journals.plos.org/plosone/s/file?id=ba62/PLOSOne_formatting_sample_title_authors_affiliations.pdf

2. Thank you for stating the following in the Acknowledgments Section of your manuscript:

“This study was financed in part by the Coordenação de Aperfeiçoamento de Pessoal de Nível Superior - Brasil (CAPES) - Finance Code 001. Grant 2022/05715-7, São Paulo Research Foundation (FAPESP). The funders had no role in the study design, data collection and analysis, decision to publish, or preparation of the manuscript.”

We note that you have provided funding information that is not currently declared in your Funding Statement. However, funding information should not appear in the Acknowledgments section or other areas of your manuscript. We will only publish funding information present in the Funding Statement section of the online submission form.

Please remove any funding-related text from the manuscript and let us know how you would like to update your Funding Statement. Currently, your Funding Statement reads as follows:

“This study was financed in part by the Coordenação de Aperfeiçoamento de Pessoal de Nível Superior - Brasil (CAPES) - https://www.gov.br/capes/pt-br - Finance Code 001 (LSO, NZF). Grant 2022/05715-7, São Paulo Research Foundation (FAPESP) - https://fapesp.br/ - (HLC). The funders had no role in the study design, data collection and analysis, decision to publish, or preparation of the manuscript.”

Please include your amended statements within your cover letter; we will change the online submission form on your behalf.

3. If the reviewer comments include a recommendation to cite specific previously published works, please review and evaluate these publications to determine whether they are relevant and should be cited. There is no requirement to cite these works unless the editor has indicated otherwise.

[Note: HTML markup is below. Please do not edit.]

Reviewers' comments:

Reviewer's Responses to Questions

**Comments to the Author**

1. Is the manuscript technically sound, and do the data support the conclusions?

Reviewer #1: Yes

Reviewer #2: Partly

**********

2. Has the statistical analysis been performed appropriately and rigorously?

Reviewer #1: Yes

Reviewer #2: Yes

**********

3. Have the authors made all data underlying the findings in their manuscript fully available?

The PLOS Data policy

Reviewer #1: Yes

Reviewer #2: Yes

**********

4. Is the manuscript presented in an intelligible fashion and written in standard English?

Reviewer #1: Yes

Reviewer #2: Yes

**********

Reviewer #1: The present study investigated the effects of eight weeks of HIIT on blood pressure and endothelial-related gene expression in aging spontaneously hypertensive rats. The authors observed improvements in exercise performance and reductions in systolic blood pressure following the intervention, accompanied by an absence of significant changes in the expression of endothelial-related genes. While the study was well-conducted and confirms known physiological benefits of HIIT, its overall novelty is limited. The improvement in blood pressure and exercise performance in hypertensive models following HIIT is well established in the literature, and the present findings corroborate existing evidence. The manuscript would be strengthened by providing mechanistic insights that extend beyond confirmation of expected outcomes. However, the current experimental design does not allow for such advancement, as no direct vascular functional measurements or mechanistic analyses were performed. The molecular assessments included do not reveal new regulatory pathways and do not sufficiently explain the observed physiological adaptations. Below are specific comments intended to help strengthen the manuscript:

Majors

The authors report that HIIT improved blood pressure and exercise capacity without altering the expression of NOS3, VEGF, EDN1, or PIK3R2. Given that these measurements were obtained after a prolonged training period, the lack of transcriptional differences suggests that chronic HIIT did not induce sustained remodeling of these pathways in the tissues examined. However, the absence of transcriptional changes does not preclude functional adaptation occurring through post-transcriptional, translational, or post-translational mechanisms. This distinction should be more clearly stated, as the current version may imply that no biologically meaningful adaptation occurred. I would suggest the authors expand their discussion by suggesting that chronic adaptations may not be reflected in skeletal muscle gene expression and that vascular or protein-level analyses would be required to fully elucidate the mechanisms underlying the observed blood pressure reduction.

Line 352. When comparing the present findings with previous studies reporting increased NOS3 expression or reduced EDN1 levels following moderate-intensity exercise (e.g., refs 46–48), the authors should acknowledge that those studies primarily assessed vascular tissues or endothelial function, rather than skeletal muscle gene expression. In contrast, the present study evaluated gene expression in soleus and diaphragm muscles. Because tissue specificity may influence transcriptional responses, the comparison between vascular-based studies and skeletal muscle mRNA measurements should be interpreted with caution. Therefore, the differences in tissue type may account for the apparent discrepancy between the current findings and prior literature, rather than implying that training intensity alone explains the divergent results.

Minors

Line 240. Please clarify which statistical test was used (e.g., one-way ANOVA or two-way ANOVA). The Statistical Analysis section refers to Tukey’s test, whereas the figure legend mentions Bonferroni. Please ensure consistency and specify which post hoc test was applied. Please specify the version of the statistical software used for analyses. Please clarify the post hoc test used to identify differences after the Kruskal-Wallis test, or remove this information if no nonparametric test was used.

Lines 332-333. Figure 8 appears to be discussed within the Discussion section; please consider relocating this figure to the Results section or adjusting the text accordingly.

Reviewer #2: The present study investigated the impact of high-intensity interval training (HIIT) on blood pressure and endothelial-related gene expression. Overall, the Introduction section requires better organization and contextualization. The discussion of NOS3, VEGF, EDN1, and related markers appears disconnected and should be better integrated to provide a clearer rationale. Additionally, the authors should explain more explicitly how high-intensity exercise may influence gene expression, particularly the genes investigated in the present study.

The Discussion section is somewhat speculative and would benefit from the inclusion of additional studies examining the effects of HIIT on blood pressure responses in animal models, particularly in rats.

Specific comments:

Lines 74–80: Please clarify whether the reported changes in gene expression are directly related to hypertension and, if so, explain the underlying rationale more clearly.

Line 85: The phrase “loss of functional integrity” is unclear. Please specify which tissue or structure is being referred to.

Line 86: Is the association between increased EDN1 and endothelial dysfunction explained solely by its regulation within the central nervous system? Please revise and clarify this statement.

Lines 101–113: It is recommended to include appropriate references to support the statements made in this paragraph.

Lines 106–107: The background information presented does not adequately support the study’s hypothesis. This section should be revised to ensure a clearer logical connection between background evidence and the proposed hypothesis.

Lines 169–170: Please briefly describe how the exercise protocol was performed to improve clarity in the Methods section.

Line 244: Please confirm whether a two-way ANOVA was used and clarify the factors included in the analysis.

Lines 322–325: Should the tissue investigated be an aortic segment rather than skeletal muscle? Please clarify and revise accordingly.

Lines 328–330: Could this phenomenon be explained by a dissociation between vascular responses (e.g., reduced blood pressure) and the gene expression measured in muscle tissue? If so, this should be acknowledged as a limitation of the study.

Line 333: Please remove the statement “Image created in BioRender.com.”

**********

what does this mean?). If published, this will include your full peer review and any attached files.

If you choose “no”, your identity will remain anonymous but your review may still be made public.

**Do you want your identity to be public for this peer review?**  For information about this choice, including consent withdrawal, please see our Privacy Policy

Reviewer #1: No

Reviewer #2: No

**********

[NOTE: If reviewer comments were submitted as an attachment file, they will be attached to this email and accessible via the submission site. Please log into your account, locate the manuscript record, and check for the action link "View Attachments". If this link does not appear, there are no attachment files.]

To ensure your figures meet our technical requirements, please review our figure guidelines: https://journals.plos.org/plosone/s/figures

You may also use PLOS’s free figure tool, NAAS, to help you prepare publication quality figures: https://journals.plos.org/plosone/s/figures#loc-tools-for-figure-preparation.

NAAS will assess whether your figures meet our technical requirements by comparing each figure against our figure specifications.

# Author comment: Author response to Decision Letter 1

**DOI:** 10.1371/journal.pone.0358696.r002

16 Jun 2026

Dear Emily Chenette

Editor-in-Chief

Plos One

June, 16, 2026.

Thank you for the opportunity to submit a revised version of our manuscript. We appreciate the time and effort that you have dedicated to providing your valuable feedback on our work. We are grateful to the reviewers for their insightful comments. We have been able to incorporate changes to reflect most of the suggestions provided by the reviewers and have highlighted the changes in the revised manuscript.

Here is a point-by-point response to the reviewers’ comments and concerns.

We look forward to hearing from you in due course.

Yours sincerely,

Dra Francis Lopes Pacagnelli

Reviewer #1:

Thank you for the opportunity to revise our manuscript. We are grateful for your careful and considered comments. We have made every attempt to respond to each (see below) and to fully address your comments in the revised manuscript. We believe these revisions have resulted in a significantly improved manuscript.

Major

1- The authors report that HIIT improved blood pressure and exercise capacity without altering the expression of NOS3, VEGF, EDN1, or PIK3R2. Given that these measurements were obtained after a prolonged training period, the lack of transcriptional differences suggests that chronic HIIT did not induce sustained remodeling of these pathways in the tissues examined. However, the absence of transcriptional changes does not preclude functional adaptation occurring through post-transcriptional, translational, or post-translational mechanisms. This distinction should be more clearly stated, as the current version may imply that no biologically meaningful adaptation occurred. I would suggest the authors expand their discussion by suggesting that chronic adaptations may not be reflected in skeletal muscle gene expression and that vascular or protein-level analyses would be required to fully elucidate the mechanisms underlying the observed blood pressure reduction.

RESPONSE: We thank the reviewer for this important and constructive comment. We agree that the absence of changes in mRNA expression does not necessarily indicate the absence of biological adaptation. In the revised manuscript, we have expanded the Discussion to clarify that exercise-induced physiological responses may occur through post-transcriptional, translational, or post-translational regulatory mechanisms that are not fully captured by gene expression analysis alone. (Discussion: Pages 17-18, Lines 413-439)

2- Line 352. When comparing the present findings with previous studies reporting increased NOS3 expression or reduced EDN1 levels following moderate-intensity exercise (e.g., refs 46–48), the authors should acknowledge that those studies primarily assessed vascular tissues or endothelial function, rather than skeletal muscle gene expression. In contrast, the present study evaluated gene expression in soleus and diaphragm muscles. Because tissue specificity may influence transcriptional responses, the comparison between vascular-based studies and skeletal muscle mRNA measurements should be interpreted with caution. Therefore, the differences in tissue type may account for the apparent discrepancy between the current findings and prior literature, rather than implying that training intensity alone explains the divergent results.

RESPONSE: We have also clarified that molecular responses to exercise may vary across tissues and biological levels of regulation. Therefore, we agree that comparisons between studies evaluating vascular function and those assessing gene expression in skeletal muscle should be interpreted with caution. (Introduction: Page 5, Lines 119-124; and Discussion: Page 17, Line 418-420).

We have indicated this aspect as a limitation of the study, highlighting that future investigations including vascular functional analyses or protein-level measurements are necessary to better elucidate the mechanisms underlying the observed reduction in blood pressure following HIIT. (Discussion: Page 19, Line 453- 459)

Minor

3- Line 240. Please clarify which statistical test was used (e.g., one-way ANOVA or two-way ANOVA). The Statistical Analysis section refers to Tukey’s test, whereas the figure legend mentions Bonferroni. Please ensure consistency and specify which post hoc test was applied. Please specify the version of the statistical software used for analyses. Please clarify the post hoc test used to identify differences after the Kruskal-Wallis test, or remove this information if no nonparametric test was used.

RESPONSE: We have clarified the statistical tests used and reformulated this description. We apologize for this unfortunate mistake. The tests have also been included in the figure legends. The version of the statistical software used has been added. (Page 11, Line 272)

4- Lines 332-333. Figure 8 appears to be discussed within the Discussion section; please consider relocating this figure to the Results section or adjusting the text accordingly.

RESPONSE: We appreciate the suggestion and have relocated Fig 8 to the end of the Results section. (Page 14, Line 346)

Reviewer #2:

Thank you for the opportunity to revise our manuscript. We are grateful for your careful and considered comments. We have made every attempt to respond to each (see below) and to fully address your comments in the revised manuscript. We believe these revisions have resulted in a significantly improved manuscript.

1- The present study investigated the impact of high-intensity interval training (HIIT) on blood pressure and endothelial-related gene expression. Overall, the Introduction section requires better organization and contextualization. The discussion of NOS3, VEGF, EDN1, and related markers appears disconnected and should be better integrated to provide a clearer rationale. Additionally, the authors should explain more explicitly how high-intensity exercise may influence gene expression, particularly the genes investigated in the present study.

RESPONSE: We thank the reviewer for this important and constructive comment. The Introduction section has been reorganized to improve contextualization. In addition, the discussion of NOS3, VEGF, EDN1, and related markers has been rewritten to provide a clearer rationale and we have added explanations of how high-intensity exercise can influence the expression of the genes under investigation. (Pages 3-5, Lines 76-108; 118-131)

2- The Discussion section is somewhat speculative and would benefit from the inclusion of additional studies examining the effects of HIIT on blood pressure responses in animal models, particularly in rats.

RESPONSE: We appreciate the suggestion. Additional studies showing the effects of HIIT on blood pressure responses in rats have been included. (Page 16, Line 388-394)

Specific comments:

3- Lines 74–80: Please clarify whether the reported changes in gene expression are directly related to hypertension and, if so, explain the underlying rationale more clearly.

RESPONSE: We hope that this information strengthens the text and helps clarify the relationship between the investigated genes and arterial hypertension. (Page 3-5, Lines 74-108)

4- Line 85: The phrase “loss of functional integrity” is unclear. Please specify which tissue or structure is being referred to.

RESPONSE: We thank the reviewer for this suggestion and have rewritten the relationships between alterations in EDN1 gene expression and their effects on endothelial dysfunction. (Page 4, Lines 96-102)

5- Line 86: Is the association between increased EDN1 and endothelial dysfunction explained solely by its regulation within the central nervous system? Please revise and clarify this statement.

RESPONSE: We thank the reviewer for this suggestion and have revised the sentence to clarify that the association between EDN1 and endothelial dysfunction is not restricted to regulation within the central nervous system. The revised text now also emphasizes that endothelin-1 is a potent vasoconstrictor peptide produced by endothelial cells and involved in the regulation of vascular tone and peripheral vascular resistance. (Page 4, Lines 96-102)

6- Lines 101–113: It is recommended to include appropriate references to support the statements made in this paragraph.

RESPONSE: We have included more references to support the statements. (Page 5, Lines 115-124)

7- Lines 106–107: The background information presented does not adequately support the study’s hypothesis. This section should be revised to ensure a clearer logical connection between background evidence and the proposed hypothesis.

RESPONSE: We thank the reviewer for this valuable suggestion. The Introduction section has been revised to improve the logical connection between the background information and the study hypothesis. Additional references have also been included to support the role of exercise in molecular signaling and angiogenic adaptations relevant to vascular function and blood pressure. (Page 5, Lines 118-131)

8- Lines 169–170: Please briefly describe how the exercise protocol was performed to improve clarity in the Methods section.

RESPONSE: The exercise protocol performed has been described. (Page 8, Lines 195-199)

9- Line 244: Please confirm whether a two-way ANOVA was used and clarify the factors included in the analysis.

RESPONSE: We have clarified the statistical test used and reformulated this description. We apologize for this unfortunate mistake. The tests have also been included in the figure legends. The version of the statistical software used has been added. (Page 11, Lines 272-275)

10- Lines 322–325: Should the tissue investigated be an aortic segment rather than skeletal muscle? Please clarify and revise accordingly.

RESPONSE: We acknowledge the reviewer’s concern. Evaluating the aorta is highly relevant in systemic arterial hypertension, and we will address this aspect as a future direction of the present study. We have presented a better justification for choosing skeletal muscle in the discussion and pointed out the study of the aorta as a perspective. (Page 15, Lines 360-364 and Page 19, Line 457)

11- Lines 328–330: Could this phenomenon be explained by a dissociation between vascular responses (e.g., reduced blood pressure) and the gene expression measured in muscle tissue? If so, this should be acknowledged as a limitation of the study.

RESPONSE: We appreciate the reviewer's concern and have adjusted the discussion accordingly, pointing out the limitations of the study. Considering that gene expression measurements were obtained after a prolonged training period, the absence of transcriptional differences suggests that chronic HIIT did not induce sustained remodeling of these pathways in the examined tissues. However, the absence of transcriptional changes does not exclude the possibility of functional adaptation through post-transcriptional, translational, or post-translational mechanisms. We have explained this more clearly in the discussion so as not to imply that no biologically significant adaptation occurred. We have also expanded the discussion, suggesting that chronic adaptations in blood pressure and functional capacity were not reflected in skeletal muscle gene expression and that vascular or protein-level analyses would be necessary to fully elucidate the mechanisms underlying the observed reduction in blood pressure. (Page 17, Lines 413-423 and Page 19, Lines 453-459)

12- Line 333: Please remove the statement “Image created in BioRender.com.”

RESPONSE: The references to the use of BioRender in Figures 1 and 8 have been removed, as suggested, to maintain conciseness and avoid unnecessary information in the figure legends. (Page 15, Line 349)

The reviewers’ observations were certainly crucial for strengthening the robustness of this manuscript, and we hope that we have adequately addressed all of their suggestions.

Attachment Submitted filename: Response to reviewers 29.04.26.docx

# Peer review: Decision Letter 1

**DOI:** 10.1371/journal.pone.0358696.r003

**Authors:** Juan M. Murias

24 Jul 2026

Dear Dr. Pacagnelli,

Please submit your revised manuscript by Sep 07 2026 11:59PM. If you will need more time than this to complete your revisions, please reply to this message or contact the journal office at plosone@plos.org. When you're ready to submit your revision, log on to https://www.editorialmanager.com/pone/ and select the 'Submissions Needing Revision' folder to locate your manuscript file.

- A letter that responds to each point raised by the academic editor and reviewer(s). You should upload this letter as a separate file labeled 'Response to Reviewers'.
- A marked-up copy of your manuscript that highlights changes made to the original version. You should upload this as a separate file labeled 'Revised Manuscript with Track Changes'.
- An unmarked version of your revised paper without tracked changes. You should upload this as a separate file labeled 'Manuscript'.

If you would like to make changes to your financial disclosure, please include your updated statement in your cover letter. Guidelines for resubmitting your figure files are available below the reviewer comments at the end of this letter.

If applicable, we recommend that you deposit your laboratory protocols in protocols.io to enhance the reproducibility of your results. Protocols.io assigns your protocol its own identifier (DOI) so that it can be cited independently in the future. For instructions see: https://journals.plos.org/plosone/s/submission-guidelines#loc-laboratory-protocols. Additionally, PLOS ONE offers an option for publishing peer-reviewed Lab Protocol articles, which describe protocols hosted on protocols.io. Read more information on sharing protocols at https://plos.org/protocols?utm_medium=editorial-email&utm_source=authorletters&utm_campaign=protocols.

As the corresponding author, your ORCID iD is verified in the submission system and will appear in the published article. PLOS supports the use of ORCID, and we encourage all coauthors to register for an ORCID iD and use it as well. Please encourage your coauthors to verify their ORCID iD within the submission system before final acceptance, as unverified ORCID iDs will not appear in the published article. *Only*  the individual author can complete the verification step; PLOS staff *cannot*  verify ORCID iDs on behalf of authors.

We look forward to receiving your revised manuscript.

Kind regards,

Juan M. Murias

Academic Editor

PLOS One

Journal Requirements:

If the reviewer comments include a recommendation to cite specific previously published works, please review and evaluate these publications to determine whether they are relevant and should be cited. There is no requirement to cite these works unless the editor has indicated otherwise.

[Note: HTML markup is below. Please do not edit.]

Reviewers' comments:

Reviewer's Responses to Questions

**Comments to the Author**

Reviewer #1: (No Response)

Reviewer #2: All comments have been addressed

**********

2. Is the manuscript technically sound, and do the data support the conclusions?

Reviewer #1: Yes

Reviewer #2: Yes

**********

3. Has the statistical analysis been performed appropriately and rigorously?

Reviewer #1: No

Reviewer #2: Yes

**********

4. Have the authors made all data underlying the findings in their manuscript fully available?

The PLOS Data policy

Reviewer #1: Yes

Reviewer #2: Yes

**********

5. Is the manuscript presented in an intelligible fashion and written in standard English?

Reviewer #1: Yes

Reviewer #2: Yes

**********

Reviewer #1: The authors have adequately addressed my previous major comments. However, I have one remaining important concern regarding the statistical analysis. In the statistical analysis section of the revised manuscript, the authors stated that distance and blood pressure were compared using t-tests. This approach is not appropriate for these outcomes because exercise capacity and systolic blood pressure were measured repeatedly over time. Exercise capacity was assessed at baseline, week 4, and week 8, while systolic blood pressure was assessed at baseline and week 8. Therefore, the analysis should account for both group and time effects.

Reviewer #2: The authors have addressed the majority of the concerns raised in my previous review, and I appreciate the substantial improvements made throughout the manuscript. I believe the manuscript is now close to being suitable for publication.

I have only a few minor comments that should be addressed before acceptance:

I recommend reporting effect sizes for the main statistical analyses, as they provide important information regarding the practical significance of the findings beyond statistical significance alone. In addition, please report the corresponding 95% confidence intervals for the effect sizes.

I noticed that some sections of the manuscript appear to have inconsistencies in font type or formatting. Please carefully review the document to ensure consistent formatting throughout the manuscript.

**********

what does this mean?). If published, this will include your full peer review and any attached files.

If you choose “no”, your identity will remain anonymous but your review may still be made public.

**Do you want your identity to be public for this peer review?**  For information about this choice, including consent withdrawal, please see our Privacy Policy

Reviewer #1: No

Reviewer #2: No

**********

[NOTE: If reviewer comments were submitted as an attachment file, they will be attached to this email and accessible via the submission site. Please log into your account, locate the manuscript record, and check for the action link "View Attachments". If this link does not appear, there are no attachment files.]

To ensure your figures meet our technical requirements, please review our figure guidelines: https://journals.plos.org/plosone/s/figures

You may also use PLOS’s free figure tool, NAAS, to help you prepare publication quality figures: https://journals.plos.org/plosone/s/figures#loc-tools-for-figure-preparation.

NAAS will assess whether your figures meet our technical requirements by comparing each figure against our figure specifications.

# Author comment: Author response to Decision Letter 2

**DOI:** 10.1371/journal.pone.0358696.r004

26 Aug 2026

Response to Reviewers

Thank you for the opportunity to revise our manuscript. We are grateful for your careful and considered comments. We have made every attempt to respond to each (see below) and to fully address your comments in the revised manuscript. We believe these revisions have resulted in a significantly improved manuscript

Reviewer #1:

“In the statistical analysis section of the revised manuscript, the authors stated that distance and blood pressure were compared using t-tests. This approach is not appropriate for these outcomes because exercise capacity and systolic blood pressure were measured repeatedly over time. Exercise capacity was assessed at baseline, week 4, and week 8, while systolic blood pressure was assessed at baseline and week 8. Therefore, the analysis should account for both group and time effects”.

Response: We thank the reviewer for this observation and agree that t-tests were not appropriate given the repeated-measures structure of these outcomes. We have re-analyzed both variables using two-way mixed-design ANOVAs (between-subjects factor: Group [Sedentary vs. HIIT]; within-subjects factor: Time), with Greenhouse-Geisser correction applied where sphericity could not be assumed.

The results were altered:

For exercise capacity, this revealed significant main effects of Group (F(1,18) = 57.78, p < .001, η²p = .76, 95% CI [.57, 1.00]) and Time (F(1.78, 32.12) = 26.96, p < .001, η²p = .60, 95% CI [.40, 1.00]), and a significant Group × Time interaction (F(1.78, 32.12) = 69.68, p < .001, η²p = .80, 95% CI [.68, 1.00]). Bonferroni-adjusted pairwise comparisons showed no significant difference between groups at baseline (p = .534), but significant divergence by Week 4 (p < .0001) and Week 8 (p < .0001), with the HIIT group showing greater exercise capacity.

For systolic blood pressure, we found a significant main effect of Group (F(1,18) = 8.68, p = .009, η²p = .33, 95% CI [.06, 1.00]), no significant main effect of Time (F(1,18) = 0.01, p = .913, η²p < .001, 95% CI [.00, 1.00]), and a significant Group × Time interaction (F(1,18) = 10.43, p = .005, η²p = .37, 95% CI [.09, 1.00]). Groups did not differ at baseline (p = .448) but differed significantly by Week 8 (p < .0001), with the HIIT group showing lower systolic blood pressure. The Statistical Analysis, Results, and Table X/Y of the revised manuscript have been updated accordingly.

Reviewer #2:

" I recommend reporting effect sizes for the main statistical analyses, as they provide important information regarding the practical significance of the findings beyond statistical significance alone. In addition, please report the corresponding 95% confidence intervals for the effect sizes."

Response: We agree and have added effect sizes with 95% confidence intervals throughout. For the ANOVA main and interaction effects, we report partial eta-squared (η²p) with 95% CIs (Table X). For pairwise group comparisons at each timepoint, we report Cohen's d with 95% CIs (Table Y). These results were added in text.

We have also extended this to the gene expression data (soleus and diaphragm NOS3, EDN1, VEGF, PIK3R2), reporting rank-biserial correlation (r) with 95% CIs alongside the Mann-Whitney U test results already used for these comparisons. The Table 1 was added in results.

Table X — ANOVA main and interaction effects, we report partial eta-squared (η²p) with 95% Cis

Outcome Effect df F p Partial η² [95% CI]

Exercise capacity Group 1.18 57.78 <0.001 0.76 [0.57, 1.00]

Time 1.78, 32.12 26.96 <0.001 0.60 [0.40, 1.00]

Group × Time 1.78, 32.12 69.68 <0.001 0.80 [0.68, 1.00]

Systolic blood pressure Group 1.18 8.68 0.009 0.33 [0.06, 1.00]

Time 1.18 0.01 0.913 <0.001 [0.00, 1.00]

Group × Time 1.18 10.43 0.005 0.37 [0.09, 1.00]

Note: η²p confidence intervals are one-sided, with the upper bound fixed at 1.00, as is standard

for variance-explained effect sizes.

Table Y. Pairwise group comparisons

Outcome Timepoint Sedentary − HIIT t df p Cohen’s d [95% CI]

Exercise capacity Baseline 32 0.63 18 0.534 0.28 [−0.74, 1.15]

Week 4 −312 −5.02 18 <0.0001 −2.25 [−5.21, −1.15]

Week 8 −731 −12.03 18 <0.0001 −5.38 [−11.3, −4.22]

Systolic blood pressure Baseline −6.8 −0.77 18 0.448 −0.35 [−1.09, 0.68]

Week 8 31.1 6.10 18 <0.0001 2.73 [2.04, 4.46]

Table 1. Group comparisons (SHR-SED vs. SHR-HIIT) for gene expression markers in soleus and diaphragm muscle.

Muscle Gene n (SED/HIIT) U p p (FDR) r [95% CI]

Soleus NOS3 10/10 73 0.089 0.313 0.46 [−0.02, 0.77]

Soleus EDN1 10/9 39 0.661 0.661 −0.13 [−0.58, 0.38]

Soleus VEGF 9/10 57 0.356 0.475 0.27 [−0.25, 0.67]

Soleus PIK3R2 10/10 67 0.218 0.348 0.34 [−0.16, 0.70]

Diaphragm NOS3 9/9 58 0.136 0.313 0.43 [−0.09, 0.77]

Diaphragm EDN1 10/10 30 0.138 0.313 −0.40 [−0.74, 0.09]

Diaphragm VEGF 9/10 27 0.156 0.313 −0.40 [−0.74, 0.11]

Diaphragm PIK3R2 10/10 43 0.631 0.661 −0.14 [−0.58, 0.36]

Mann-Whitney U test; effect sizes are reported as rank-biserial correlation (r) with 95% confidence intervals. p-values were adjusted for multiple comparisons using the Benjamini-Hochberg false discovery rate (FDR) procedure.

Comment 3: "...inconsistencies in font type or formatting. Please carefully review the document..."

Response: We have reviewed the manuscript in full and corrected the font and formatting inconsistencies throughout.

Attachment Submitted filename: Response to reviewers 25.08.2026.docx

# Peer review: Decision Letter 2

**DOI:** 10.1371/journal.pone.0358696.r005

**Authors:** Juan M. Murias

3 Sep 2026

<p>Endothelial-related gene expression profile of muscle adaptation to high-intensity interval training in arterial hypertension

PONE-D-25-67337R2

Dear Dr. Pacagnelli,

We’re pleased to inform you that your manuscript has been judged scientifically suitable for publication and will be formally accepted for publication once it meets all outstanding technical requirements.

Within one week, you’ll receive an e-mail detailing the required amendments. When these have been addressed, you’ll receive a formal acceptance letter and your manuscript will be scheduled for publication.

An invoice will be generated when your article is formally accepted. Please note, if your institution has a publishing partnership with PLOS and your article meets the relevant criteria, all or part of your publication costs will be covered. Please make sure your user information is up-to-date by logging into Editorial Manager at Editorial Manager® and clicking the ‘Update My Information' link at the top of the page. For questions related to billing, please contact billing support.

If your institution or institutions have a press office, please notify them about your upcoming paper to help maximize its impact. If they’ll be preparing press materials, please inform our press team as soon as possible -- no later than 48 hours after receiving the formal acceptance. Your manuscript will remain under strict press embargo until 2 pm Eastern Time on the date of publication. For more information, please contact onepress@plos.org.

Kind regards,

Juan M. Murias

Academic Editor

PLOS One

Additional Editor Comments (optional):

Reviewers' comments:

Reviewer's Responses to Questions

**Comments to the Author**

Reviewer #1: All comments have been addressed

**********

2. Is the manuscript technically sound, and do the data support the conclusions?

Reviewer #1: Yes

**********

3. Has the statistical analysis been performed appropriately and rigorously?

Reviewer #1: Yes

**********

4. Have the authors made all data underlying the findings in their manuscript fully available?

The PLOS Data policy

Reviewer #1: Yes

**********

5. Is the manuscript presented in an intelligible fashion and written in standard English?

Reviewer #1: Yes

**********

Reviewer #1: The authors have adequately addressed my remaining concern. I have no further comments on the manuscript.

**********

what does this mean?). If published, this will include your full peer review and any attached files.

If you choose “no”, your identity will remain anonymous but your review may still be made public.

**Do you want your identity to be public for this peer review?**  For information about this choice, including consent withdrawal, please see our Privacy Policy

Reviewer #1: No

**********

# Editor report: Acceptance letter

**DOI:** 10.1371/journal.pone.0358696.r006

**Authors:** Juan M. Murias

PONE-D-25-67337R2

PLOS One

Dear Dr. Pacagnelli,

I'm pleased to inform you that your manuscript has been deemed suitable for publication in PLOS One. Congratulations! Your manuscript is now being handed over to our production team.

At this stage, our production department will prepare your paper for publication. This includes ensuring the following:

* All references, tables, and figures are properly cited

* All relevant supporting information is included in the manuscript submission,

* There are no issues that prevent the paper from being properly typeset

You will receive further instructions from the production team, including instructions on how to review your proof when it is ready. Please keep in mind that we are working through a large volume of accepted articles, so please give us a few days to review your paper and let you know the next and final steps.

Lastly, if your institution or institutions have a press office, please let them know about your upcoming paper now to help maximize its impact. If they'll be preparing press materials, please inform our press team within the next 48 hours. Your manuscript will remain under strict press embargo until 2 pm Eastern Time on the date of publication. For more information, please contact onepress@plos.org.

You will receive an invoice from PLOS for your publication fee after your manuscript has reached the completed accept phase. If you receive an email requesting payment before acceptance or for any other service, this may be a phishing scheme. Learn how to identify phishing emails and protect your accounts at https://explore.plos.org/phishing.

If we can help with anything else, please email us at customercare@plos.org.

Thank you for submitting your work to PLOS One and supporting open access.

Kind regards,

PLOS One Editorial Office Staff

on behalf of

Dr. Juan M. Murias

Academic Editor

PLOS One
